# Discovery and Characterization of the Key Constituents in *Ginkgo biloba* Leaf Extract With Potent Inhibitory Effects on Human UDP-Glucuronosyltransferase 1A1

**DOI:** 10.3389/fphar.2022.815235

**Published:** 2022-02-21

**Authors:** Hui-Lin Pang, Guang-Hao Zhu, Qi-Hang Zhou, Chun-Zhi Ai, Ya-Di Zhu, Ping Wang, Tong-Yi Dou, Yang-Liu Xia, Hong Ma, Guang-Bo Ge

**Affiliations:** ^1^ School of Life and Pharmaceutical Sciences, Dalian University of Technology, Panjin, China; ^2^ Shanghai Frontiers Science Center for Chinese Medicine Chemical Biology, Institute of Interdisciplinary Integrative Medicine Research, Shanghai University of Traditional Chinese Medicine, Shangha, China; ^3^ State Key Laboratory for Chemistry and Molecular Engineering of Medicinal Resources, School of Chemistry and Pharmacy, Guangxi Normal University, Guilin, China; ^4^ Shanghai Research Institute of Acupuncture and Meridian, Shanghai University of Traditional Chinese Medicine, Shanghai, China

**Keywords:** human UDP-glucuronosyltransferase 1A1, cell-based fluorescence assay, *Ginkgo biloba* leaves, bioflavonoids, herb-drug interactions

## Abstract

Human UDP-glucuronosyltransferase 1A1 (hUGT1A1) is one of the most essential phase II enzymes in humans. Dysfunction or strong inhibition of hUGT1A1 may result in hyperbilirubinaemia and clinically relevant drug/herb-drug interactions (DDIs/HDIs). Recently, a high-throughput fluorescence-based assay was constructed by us to find the compounds/herbal extracts with strong inhibition against intracellular hUGT1A1. Following screening of over one hundred of herbal products, the extract of *Ginkgo biloba* leaves (GBL) displayed the most potent hUGT1A1 inhibition in HeLa-UGT1A1 cells (Hela cells overexpressed hUGT1A1). Further investigations demonstrated that four biflavones including bilobetin, isoginkgetin, sciadopitysin and ginkgetin, are key constituents responsible for hUGT1A1 inhibition in living cells. These biflavones potently inhibit hUGT1A1 in both human liver microsomes (HLM) and living cells, with the IC_50_ values ranging from 0.075 to 0.41 μM in living cells. Inhibition kinetic analyses and docking simulations suggested that four tested biflavones potently inhibit hUGT1A1-catalyzed NHPN-*O*-glucuronidation in HLM *via* a mixed inhibition manner, showing the *K*_*i*_ values ranging from 0.07 to 0.74 μM. Collectively, our findings uncover the key constituents in GBL responsible for hUGT1A1 inhibition and decipher their inhibitory mechanisms against hUGT1A1, which will be very helpful for guiding the rational use of GBL-related herbal products in clinical settings.

## Introduction

Human UDP-glucuronosyltransferases1A1 (hUGT1A1) is one of the most essential enzymes responsible for the biotransformation and detoxification of the endogenous toxins (e.g., bilirubin), and for the metabolic elimination of numerous therapeutic and diet-derived xenobiotics (Ouzzine et al., 2003; Kiang et al., 2005). In adults, hUGT1A1 is predominantly expressed in the liver, while the conjugative enzyme is also distributed in the small intestine and kidney with relatively abundant levels (Drozdzik et al., 2018). Increasing evidence has illustrated that dysfunction or potent inhibition of hepatic UGT1A1 may trigger metabolic disorder of bilirubin, resulting in varying degrees of hyperbilirubinemia, liver disorders, and even death (Vítek and Ostrow, 2009; Bartlett and Gourley, 2011; Zhou et al., 2011; Ma et al., 2013). Furthermore, partial or complete loss of hepatic and intestinal UGT1A1 activity may affect the pharmacokinetic behaviours of the UGT1A1-substrate drugs, which in turn, enhance the *in vivo* effects of UGT1A1-substrate drugs or show clinically relevant drug/herb-drug interactions (Kadakol et al., 2000; Kiang et al., 2005) (DDI/HDI). Therefore, the major regulatory agencies including the US Food and Drug Administration (FDA) has recommended to assess the inhibition potency of new drug candidates or phytochemical products against hUGT1A1 before getting approval for marketing (US Food and Drug Administration, 2012). It also should be noted that in some cases, the UGT1A1 inhibitors with improved safety profiles can be used to increase the plasma exposure of phenolic drugs and to achieve desired *in vivo* therapeutic effects, by reducing the first pass metabolism mediated by intestinal UGT1A1 (Liu D. et al., 2019).

Over the past few decades, a wide range of hUGT1A1 inhibitors including therapeutic drugs (such as tyrosine kinase inhibitors) and herbal constituents (such as flavonoids and phenolic acids) have been reported (Zucker et al., 2001; Singer et al., 2007; Peer et al., 2012; Lv et al., 2019Liu et al., 2019). Notably, only a small part of the known hUGT1A1 inhibitors have been reported with the high risk to cause hyperbilirubinaemia or clinically relevant drug/herb-drug interactions. However, in most cases, the *in vitro* hUGT1A1 inhibition results do not match to the *in vivo* hUGT1A1 inhibition potency. Especially, many herbal constituents have been found with strong inhibition potentials against hUGT1A1, but in most cases, the inhibition potentials of these natural products in living systems are extremely weak (Goon et al., 2016). The primary cause of the false positives in hUGT1A1 inhibition assays is the enzyme sources. Currently almost all hUGT1A1 inhibition assays were performed by using recombinant hUGT1A1 or human liver preparations as enzyme sources. Under such incubation conditions, the small molecule inhibitors can directly bind on the target enzyme, lacking the physical barrier of cell membrane. It is well-known that hUGT1A1 is located within the lumen of endoplasmic reticulum. In this regard, the hUGT1A1 inhibitors should be able to penetrate the cell membrane to bind the target enzyme in the living cells. Thus, it is important to use a more practical and convenient approach for screening hUGT1A1 inhibitors to avoid the high false positive rate.

Recently, a high-throughput cell-based fluorescence assay was developed by using an artificial specific fluorescence substrate (termed N-butyl-4-(4-hydroxyphenyl)-1,8-naphthalimide (NHPN)) as the probe substrate and the hUGT1A1 overexpressed Hela cells (HeLa-UGT1A1 cells) as the enzyme sources (Zhu et al., 2020). With the help of this newly designed cell-based hUGT1A1 inhibition assay, we have screened the inhibition potentials of a number of commonly used herbal medicines against hUGT1A1 in living cells. The results clearly demonstrated that the used extract of *Ginkgo biloba* leaves (GBL) displayed the most potent hUGT1A1 inhibition potency, with the inhibition rate exceeding 95% at the dose of 100 μg/ml. This finding encouraged us to further investigate the inhibition potentials of GBL and its major constituents against hUGT1A1 in various enzyme sources including HeLa-UGT1A1 cells and human liver microsomes, as well as to identify the key constituents in GBL responsible for hUGT1A1 inhibition. Furthermore, the inhibition potential and the inhibitory mechanisms of the newly identified naturally occurring hUGT1A1 inhibitors from GBL were also investigated by performing an array of inhibition kinetic assays and docking simulations. All these studies are very helpful for researchers to understand the interactions between the used GBL and human drug-metabolizing enzymes, and also provide the key data to assess potential risks or beneficial effects of the used GBL by inhibition of hUGT1A1.

## Materials and Methods

### Chemicals and Reagents

The standard *Ginkgo biloba* leaf extract (GBL) was obtained from Shanghai Sine Promod Pharmaceutical Co., Ltd. (Shanghai, China), and the chemical analysis of *Ginkgo biloba* leaf extract was analyzed by using ultra high performance liquid Chromatography-Q exactive hybrid quadrupole orbitrap high-resolution accurate mass spectrometric (UHPLC-Q-Orbitrap HRMS, Thermo Fisher Scientific Inc., Grand Island, NY, United States) (Supplementary Figure S2 and Supplementary Table S2). Other 126 herbal products were provided by Tianjiang Pharmaceutical Co., Ltd (Jiangsu, China) (Supplementary Table S1). N-butyl-4-(4-hydroxyphenyl)-1,8-naphthalimide (NHPN) and NHPN-*O*-glucuronide (NHPNG) were chemically synthesized by one of the authors (Ping Wang) according to the previously reported scheme (Lv et al., 2017), with the purities ≥98%. Sixteen chemical constituents in GBL extract were purchased from Chengdu Gelipu Biotechnology Co., Ltd. (Chengdu, Sichuan, China), with the purities ≥96%. Human liver microsomes (HLMs) (from 50 donors, lot no. X008067) were obtained from Bioreclamation IVT (Baltimore, MD, United States). Recombinant UGT1A1 enzymes were purchased from BD Gentest Corp (Woburn, MA, United States). Tris and HCl were acquired from Sinopharm Chemical Reagent Co. Ltd. (Shanghai, China) and MgCl_2_ was obtained from Meilunbio (Dalian, China). UDPGA trisodium salt and polyethylene glycol hexadecyl ether (Brij 58) were purchased from Sigma-Aldrich (St. Louis, MO, United States). Cell culture medium and fetal bovine serum were obtained from Hylcone (Logan, UK). Phosphate buffered saline (0.1 M, pH = 7.4), Millipore water and LC grade organic solvent (including acetonitrile, methanol and formic acid) were ordered from Tedia company (Tedia, United States) and used for all experiments.

### Development and Validation of HeLa1A1 Cells

The UGT1A1-overexpressing HeLa cells were constructed according to the previous studies (Zhang et al., 2015; Zhang et al., 2019; Zhu et al., 2020). Briefly, UGT1A1 cDNA was synthesized and subsequently subcloned into the BamHI and MluI sites of the pLVXmCMV-ZsGreen-PGK-Puro vector. Next, lentiviral vectors were produced by transient transfection into 293T cells based on the third-generation packaging system. After that, wild-type HeLa cells were transfected following incubation with the lentivirus. The optimal value was obtained when the cells were best transfected as published previously (Zhang et al., 2015; Zhang et al., 2019; Zhu et al., 2020). The stably transfected HeLa cells were named as HeLa-UGT1A1 cells. The protein levels of hUGT1A1 in HeLa-UGT1A1 cells were assayed by Western blotting, while UGT1A1-catalyzed *O*-glucuronidation activity of HeLa-UGT1A1 cells were determined by using NHPN as the probe substrate (Supplementary Figure S1).

### Determination of NHPN and NHPNG Using LC-FD

A RF-20A FD detector coupled UFLC system (Shimadzu, Kyoto, Japan), equipped with a DGU-20A5R vacuum degasser, two LC-20ADXR pumps, a SIL-20ACXR autosampler and a CTO-20A column oven, was used to quantify NHPN and NHPNG. Chromatographic separation of analytes was carried out on a shim-pack Acchrom HC-C18 analytical column (2.0 × 150 mm, 4.6 μm particle size), while the column temperature was kept at 40°C. The flow rate was kept at 0.5 ml/min. The mobile phase consisted of acetonitrile (A) and water containing 0.2% formic acid (B). The gradient of the mobile phase was as follows: 0–0.5 min, 80–60% B; 0.5–3.5 min, 60–5% B; 3.5–5 min, 5% B; 5–7.5 min, equilibration with 80% B. The excitation wavelength and the emission wavelength were set at 370 and 520 nm, respectively for detection.

### UGT1A1 Inhibition Assays

#### UGT1A1 Inhibition Assay in HeLa-UGT1A1 Cells

The inhibitory effects of herbal products and chemical constituents of GBL extract on UGT1A1 were investigated in living HeLa-UGT1A1 cells by using a specific fluorescent substrate, NHPN, as a probe. HeLa-UGT1A1 cells were cultured at 37°C in 5% CO_2_ in Modified Eagle’s Medium (MEM) containing 0.1% antibiotic-antimycoticmix, supplemented with 10% fetal bovine serum (FBS). HeLa-UGT1A1 cells were seeded in 96-well plates and cultured for 24 h. The cells were incubated in the medium containing different kinds of herbal products (final concentration, 10 μg/ml) or chemical constituents of GBL extract (diluted to different concentration by DMSO with the final concentration of 1% DMSO in each incubation) for 60 min at 37°C under 5% CO_2_, and co-incubated with NHPN (final concentration, 50 μM) for another 60 min. The reaction was terminated by adding an equal volume of ice-cold acetonitrile to each well, and then centrifuged at 20,000×*g* for 20 min at 4°C. After centrifugation, 5 μl aliquots of the supernatant was taken for LC-FD analysis. The validation data for this cell-based flourescence assay can be found in our previous publication (Zhu et al., 2020).

#### UGT1A1 Inhibition Assay in HLM and Recombinant UGT1A1

NHPN was used for evaluating the inhibitory effects of GBL extract on HLM. A typical incubation mixture with a total volume of 200 μl consisted of 50 mM Tris-HCl (pH = 7.4), 5 mM MgCl_2_, 2 mM UDPGA, NHPN (diluted by DMSO, 5 μM) and the enzymes. Inhibitors (ginkgetin, bilobetin, isoginkgetin, sciadopitysin) were serially diluted to the required concentration by DMSO. The final concentration of DMSO in the incubation mixture was less than 1%. When HLM was used as the enzyme source, HLM (10 μg/ml) and Brij 58 (0.1 mg/mg microsomal protein) were pre-incubated for 20 min at 4°C to disrupt the ER membrane to remove the latency. A concentration of 5 μg/ml was used when recombinant UGT1A1 was the enzyme source. After pre-incubation for 3 min at 37°C, UDPGA was added to the incubation mixture to initiate the reaction. After 60 min incubation at 37°C, the reaction was terminated by adding 200 μl ice-cold acetonitrile, and the incubation mixture was kept on ice. After centrifugation at 20,000×*g* for 20 min at 4°C, 5 μl aliquots of the supernatant was taken for LC-FD analysis.

The residual activity of hUGT1A1 was determined as follows: Residual activity (%) = (the fluorescence intensity of NHPNG generated in the incubations with inhibitor)/(the fluorescence intensity of NHPNG generated in the incubations without inhibitor) × 100%. A set of incubations with the same fluorescent substrate concentration and different inhibitor concentrations were performed to determine the half maximal inhibition concentration (IC_50_).

### Inhibition Kinetic Analyses

A set of incubations by changing both the fluorescent substrate concentration and the inhibitor concentration were performed to determine the inhibition kinetic modes and the corresponding inhibition constant (*K*_*i*_) values. The incubation and analytical sample preparation were conducted as described previously. Goodness-of-fit parameter and Akaike’s Information Criterion (AIC) were employed to select the most appropriate inhibition kinetic modes from the following three inhibition modes including competitive inhibition Eq. 1, noncompetitive inhibition Eq. 2, or mixed inhibition Eq. 3,

$$V=\frac{V_{\max}\times \left[S\right]}{K_{m}\times \left(1+\frac{\left[I\right]}{K_{i}}\right)+\left[S\right]}$$ (1)

$$V=\frac{V_{\max}\times \left[S\right]}{\left(K_{m}+\left[S\right]\right)\times \left(1+\frac{\left[I\right]}{K_{i}}\right)}$$ (2)

$$V=\frac{V_{\max}\times \left[S\right]}{K_{m}\times \left(1+\frac{\left[I\right]}{K_{i}}\right)+\left[S\right]\times \left(1+\frac{\left[I\right]}{\alpha K_{i}}\right)}$$ (3)

Here, *V* is the turnover of the glucuronidation reaction; *V*_*max*_ is the maximum velocity; *S* and *I* are the substrate and inhibitor concentrations, respectively; *K*_*m*_ is the Michaelis constant (substrate concentration at 0.5 *V*_*max*_); *K*_*i*_ is the inhibition constant that describes the affinity of the inhibitor towards hUGT1A1. The corresponding *K*_*i*_ value of each tested inhibitor is calculated by utilizing the slopes of second plot from Lineweaver-Burk plot versus inhibitor concentrations.

### Molecular Docking Simulations

Docking simulations were used to explore the binding modes of each inhibitor into hUGT1A1 by using AutoDock Vina (1.1.2) (Luo et al., 2008). The 3D structure of hUGT1A1 was recently predicted by the AlphaFold (UniProt code: P22309) (Luo et al., 2008). Firstly, the structure of UDPGA was docked into the catalytic domain of hUGT1A1 by the alignment function of PyMOL (The PyMOL Molecular Graphics System 2.4.0a0 Open-Source, Schrödinger LLC., New York, United States) according to the structure of UGT74AC1 mutant (PDB code: 6L8Z) (Li et al., 2020). Secondly, the structures of target protein (hUGT1A1) and each ligand (including NHPN and each bioflavone-type inhibitor) were preprocessed by AutoDockTools 1.5.6, including adding polar hydrogen atoms, calculating atomic charges and defining atom types. Then, the druggable pockets of hUGT1A1 predicted by CavityPlus were defined as the ligand-binding sites (Xu et al., 2018; Li et al., 2020), which were used to dock each tested inhibitor. Meanwhile, the substrate NHPN was docked into the catalytic site of hUGT1A1 in the presence of UDPGA. Finally, receptor-ligand interactions analysis of the top docking poses was conducted by Discovery Studio Visualizer (BIOVIA Discovery Studio 2019; Dassault Systèmes, San Diego, United States).

### Data Analysis

All assays were conducted in triplicate. Data was expressed as mean ± SD. IC_50_ and *K*_*i*_ values were determined by using GraphPad Prism 7.0 (GraphPad Software, Inc., La Jolla, United States).

## Results

### Screening of the Herbal Products With Strong UGT1A1 Inhibition Potency

**FIGURE 1:**
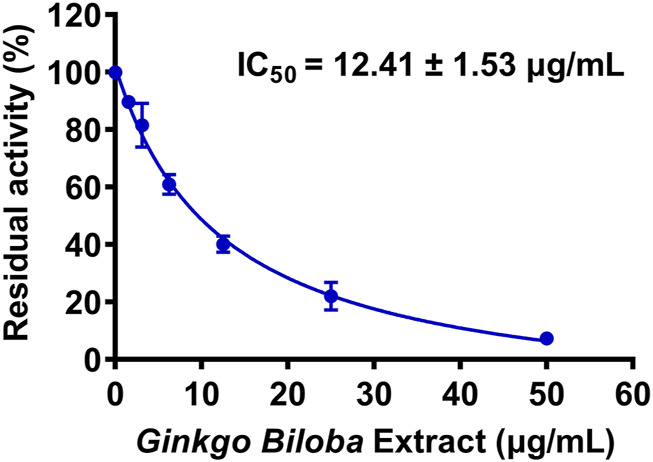
Dose-inhibition curve of *Ginkgo biloba* leaf extract on UGT1A1-catalyzed NHPN-*O*-glucuronidation in HeLa-UGT1A1 cells. The data were expressed as the means of triplicate determinations.

First, 127 herbal products were collected and their inhibitory effects on hUGT1A1 were assayed in HeLa-UGT1A1 cells at the final concentration of 10 μg/ml. As shown in Supplementary Table S1, among all tested herbal products, GBL displayed the most potent hUGT1A1 inhibition in living cells, with the residual activity of hUGT1A1 was less than 40%. To quantify the inhibition potency of GBL, the dose-dependent inhibition curve of GBL against hUGT1A1 were plotted by utilizing increasing concentrations of GBL. As depicted in Figure 1, the standard extract of GBL dose-dependently inhibits hUGT1A1-mediated NHPN*-O-*glucuronidation in HeLa-UGT1A1 cells, with an apparent IC_50_ value as low as 12.41 μg/ml.

### Inhibition of hUGT1A1 by the Constituents in GBL in Living Cells

**FIGURE 2:**
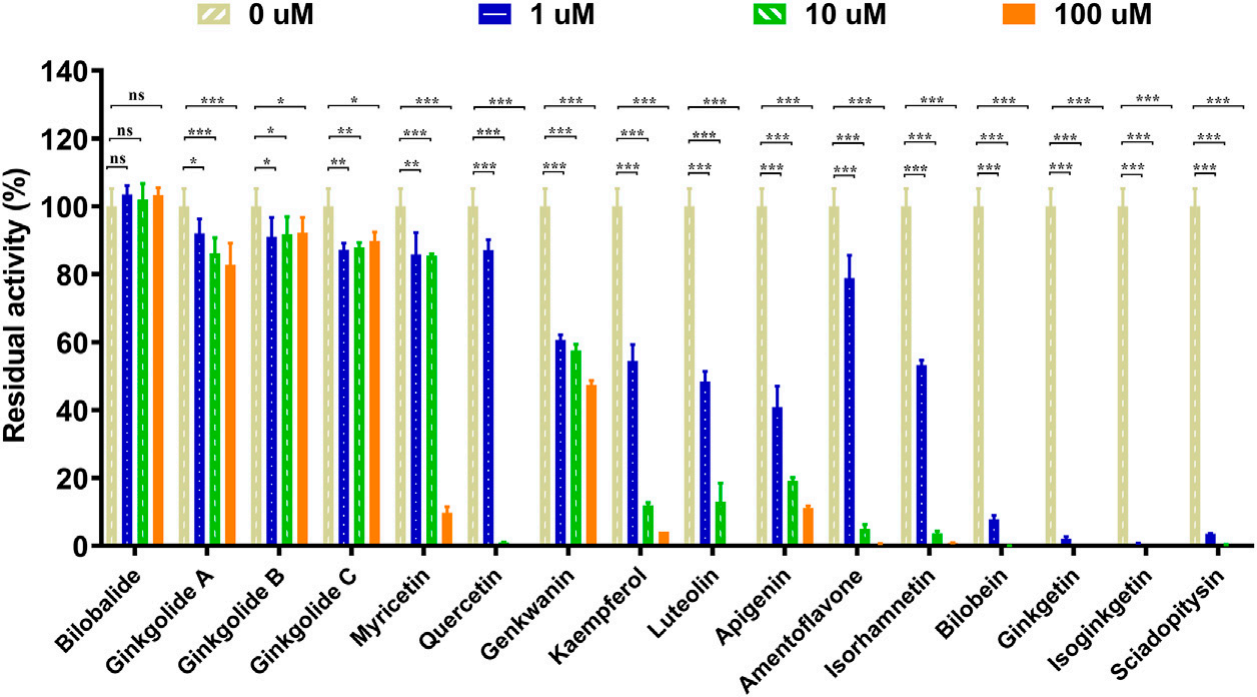
Inhibitory effects of chemical constituents of *Ginkgo biloba* leaf extract (final concentration, 1, 10, 100 μM) on UGT1A1-catalyzed NHPN-O-glucuronidation in HeLa-UGT1A1 cells. The data were expressed as the means of triplicate determinations. ns, no significance; *p < 0.05; **p < 0.01; ***p < 0.001.

**FIGURE 3:**
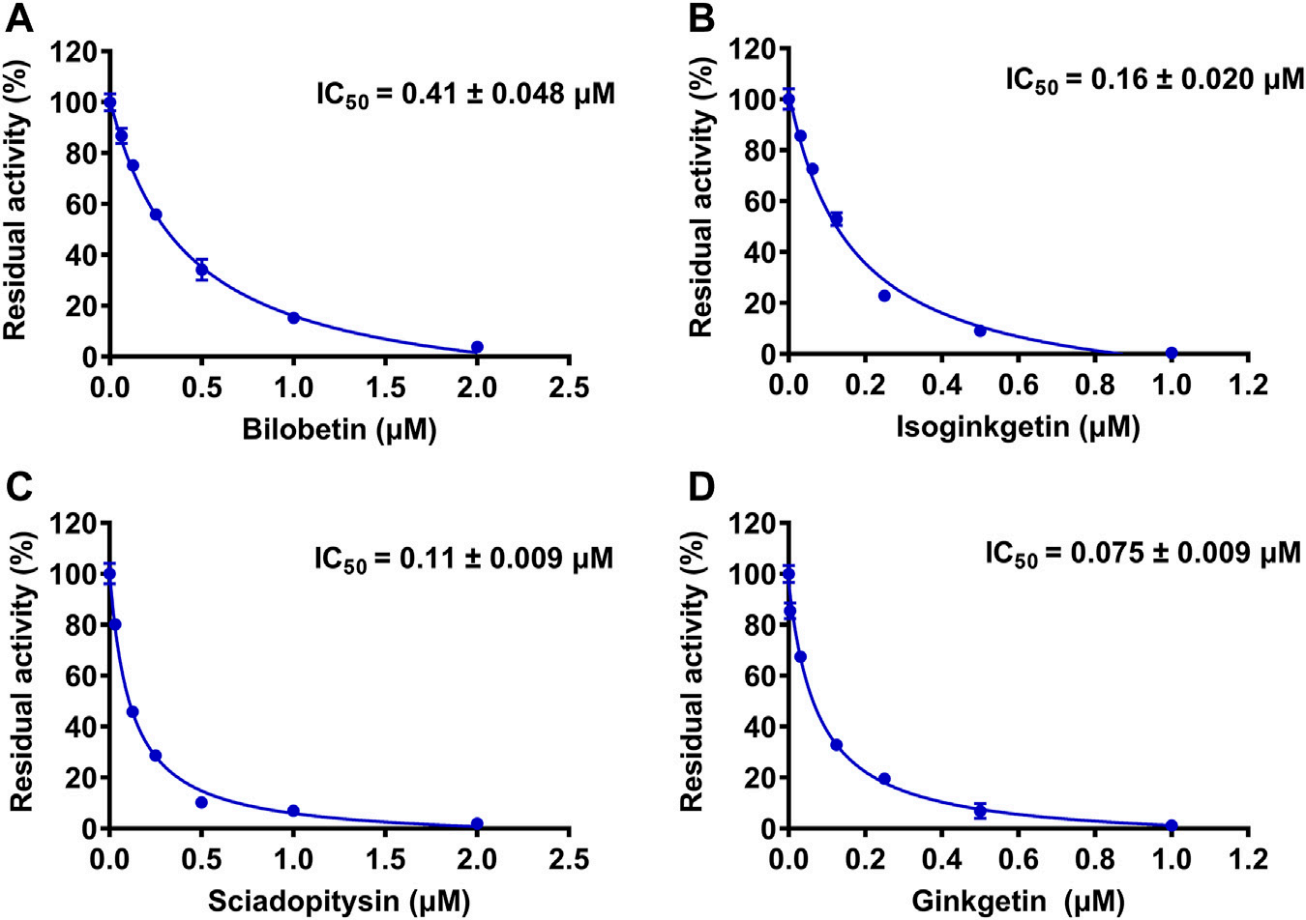
The dose-dependent inhibition curves of bilobetin **(A)**, isoginkgetin **(B)**, sciadopitysin **(C)** and ginkfetin **(D)** on NHPN-*O*-glucuronidation in HeLa-UGT1A1 cells. The data were expressed as the means of triplicate determinations.

The chemical constituents in GBL were investigated by using ultra high performance liquid Chromatography-Q exactive hybrid quadrupole orbitrap high-resolution accurate mass spectrometric (UHPLC-Q-Orbitrap HRMS) (Supplementary Figure S2). A total of 51 chemicals were identified or tentatively characterized from GBL (Supplementary Table S2), including 32 flavonol glycosides, 5 terpene trilactones, 5 biflavones, 3 flavanols, 4 flavonols, and 2 organic acids, although some compounds which do not ionize in negative mode may not be detected. To discover the key constituents in GBL responsible for hUGT1A1 inhibition, sixteen representative constituents (including seven flavonoids, five bioflavones and four terpene lactones) present in GBL extract, were purchased for assaying their hUGT1A1 inhibition potentials in Hela-UGT1A1 cells. As shown in Figure 2 and Table 1, the inhibition potency of 16 constituents in GBL varied significantly from each other. Four terpene lactones (bilobalide, ginkgolide A, ginkgolide B, and ginkgolide C) did not inhibit intracellular hUGT1A1 even at high concentration (100 μM). By contrast, seven tested flavonoids (myricetin, quercetin, apigenin, isorhamnetin, luteolin, genkwanin and kaempferol) displayed moderate to weak inhibition against intracellular hUGT1A1, with the IC_50_ values ranging from 1.38 μM (kaempferol) to 64.56 μM (myricetin) (Supplementary Figure S3). Notably, five tested bioflavones (amentoflavone, bilobetin, isoginkgetin, sciadopitysin and ginkgetin) displayed strong hUGT1A1 inhibition potency in living cells. In particular, bilobetin, isoginkgetin, sciadopitysin and ginkgetin dose-dependently inhibits hUGT1A1-catalyzed NHPN*-O-*glucuronidation in HeLa-UGT1A1 cells, with the apparent IC_50_ values ranging from 0.075 μM (ginkgetin) to 0.41 μM (bilobetin) (Figure 3). The five biflavones were quantified to be 5.89, 12.07, 7.06, 12.78, and 1.20 μg/g for amentoflavone, bilobetin, ginkgetin, isoginkgetin and sciadopitysin, respectively, in the used GBL using HPLC-UV (see Supplementary Material), which corresponds to a content in p.p.m. range.

**TABLE 1:** The IC_50_ valuses of chemical constituents of *ginkgo biloba* extract on hUGT1A1-catalyzed NHPN-*O*-glucuronidation in HeLa-UGT1A1 cells.

No	Compound	CAS	MW	Enzyme source	IC_50_ (μM)
1	Myricetin	529-44–2	318.23	HeLa-UGT1A1	64.56 ± 12.24
2	Quercetin	117-39–5	302.24	HeLa-UGT1A1	2.05 ± 0.24
3	Apigenin	520-36–5	270.24	HeLa-UGT1A1	1.89 ± 0.24
4	Lsorhamnetin	480-19–3	316.26	HeLa-UGT1A1	1.68 ± 0.34
5	Luteolin	491-70–3	286.24	HeLa-UGT1A1	1.41 ± 0.17
6	Genkwanin	437-64–9	284.26	HeLa-UGT1A1	1.50 ± 0.46
7	Kaempferol	520-18–3	286.24	HeLa-UGT1A1	1.38 ± 0.19
8	Amentoflavone	1,617-53–4	538.46	HeLa-UGT1A1	3.29 ± 0.47
9	Bilobetin	521-32–4	552.50	HeLa-UGT1A1	0.41 ± 0.048
10	Isoginkgetin	548-19–6	566.51	HeLa-UGT1A1	0.16 ± 0.020
11	Sciadopitysin	521-34–6	580.55	HeLa-UGT1A1	0.11 ± 0.009
12	Ginkgetin	481-46–9	566.52	HeLa-UGT1A1	0.075 ± 0.009
13	Bilobalide	33,570-04–6	326.30	HeLa-UGT1A1	>100
14	Ginkgolide A	15,291-75–5	408.40	HeLa-UGT1A1	>100
15	Ginkgolide B	15,291-76–6	440.40	HeLa-UGT1A1	>100
16	Ginkgolide C	15,291-77–7	424.40	HeLa-UGT1A1	>100

### Inhibition Kinetic Analyses of GBL and the Presented Biflavones Against hUGT1A1

**FIGURE 4:**
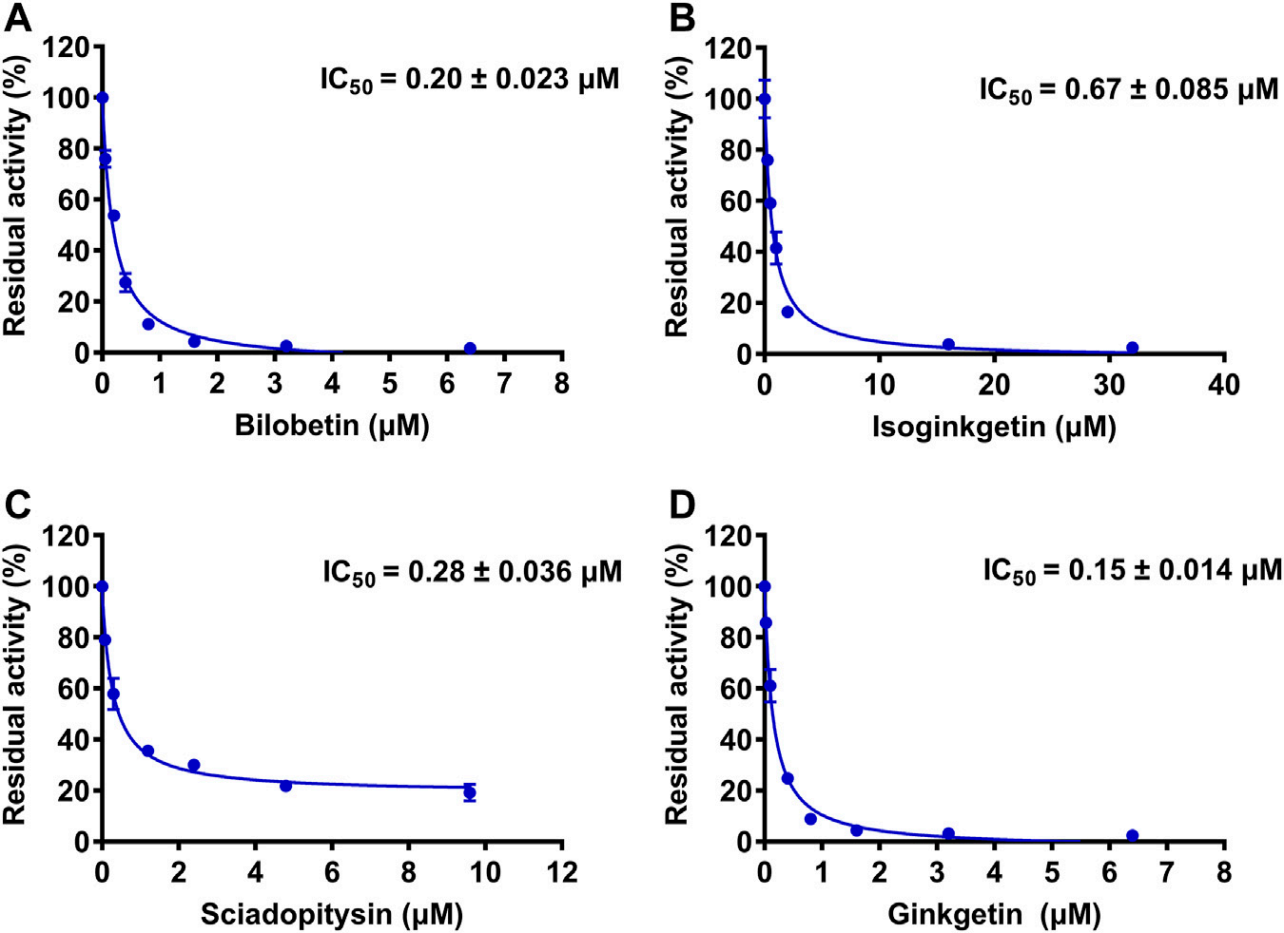
The dose-dependent inhibition curves of isoginkgetin **(A)**, sciadopitysin **(B)**, bilobetin **(C)** and ginkfetin **(D)** on NHPN-O-glucuronidation in HLM. The data were expressed as the means of triplicate determinations.

**FIGURE 5:**
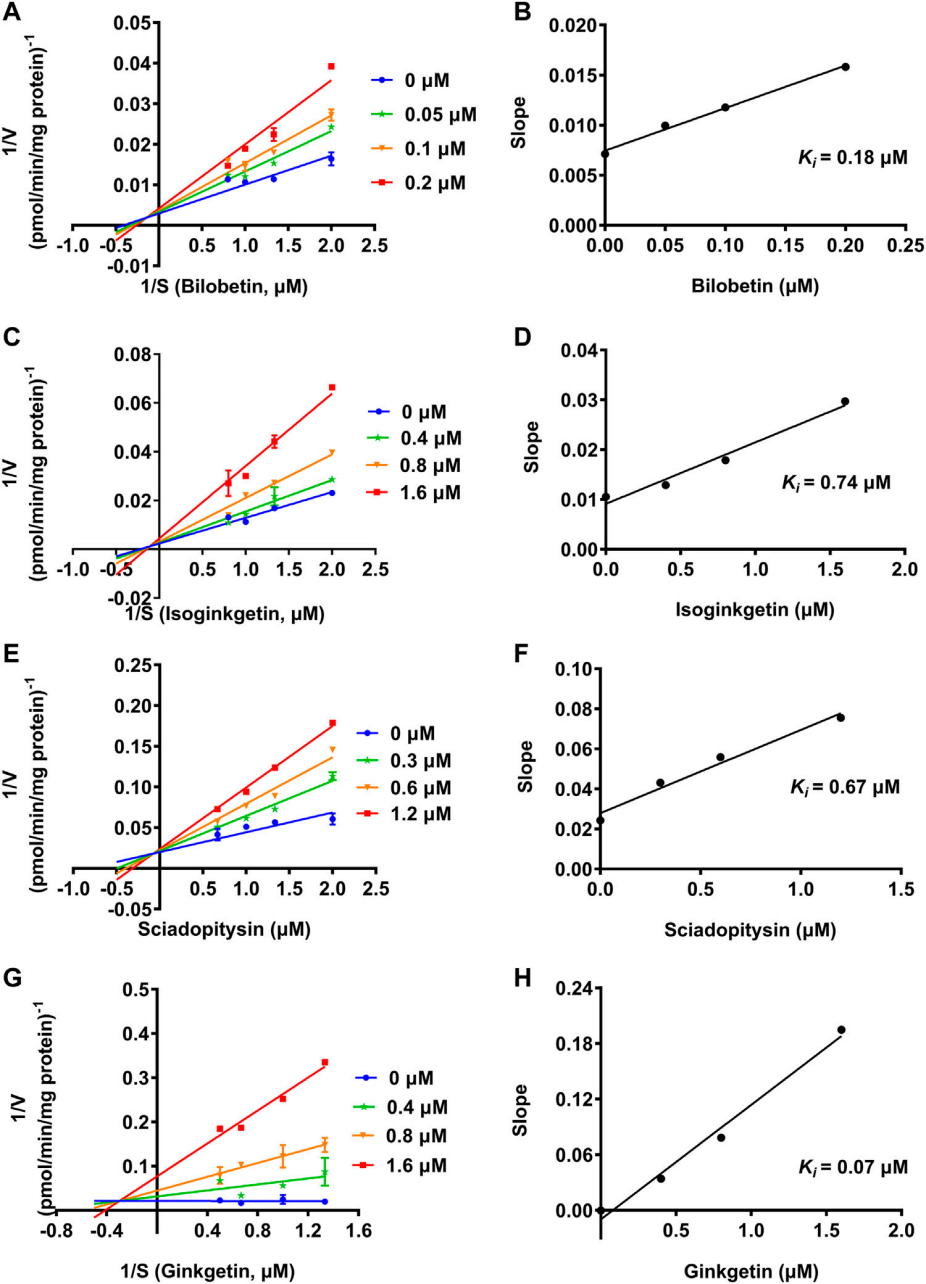
Lineweaver-Burk plots **(A, C, E, G)** of bilobetin, isoginkgetin, sciadopitysin and ginkgetin against UGT1A1-catalyzed NHPN-*O*-glucuronidation in HLM, and the second plots **(B, D, F, H)** using the slopes obtained from the Lineweaver-Burk plots *VS* the concentrations of each inhibitor.

To further explore the inhibitory mechanisms of the biflavones in GBL against hUGT1A1, a set of inhibition kinetic assays were performed by using HLM as enzyme source. As shown in Figure 4 and Table 2, bilobetin, isoginkgetin, sciadopitysin and ginkgetin also potently and dose-dependently inhibit hUGT1A1-catalzyed NHPN*-O-*glucuronidation in HLM, with the IC_50_ values ranging from 0.15 to 0.67 μM. Notably, the inhibition potentials of four tested bioflavones against hUGT1A1 in HLM are similar to that in Hela-UGT1A1 cells. However, the IC_50_ value of amentoflavone in HLM is much lower than that in living cells (0.41 vs. 3.29 μM) (Supplementary Figure S5 and Table 2). For GBL, similar IC_50_ values are observed in HLM (16.91 μg/ml) and in Hela-UGT1A1 cells (12.41 μg/ml) (Supplementary Figure S5 and Supplementary Table S2). In addition, comparable IC_50_ values are also obtained for bilobetin, isoginkgetin, sciadopitysin and ginkgetin in the recombinant UGT1A1 systems, ranging from 0.10 to 0.29 μM, to those derived from HLM (0.15–0.67 μM) (Supplementary Figure S6). The inhibition kinetic analyses of these four biflavones are further determined using HLM. As shown in Figure 5 and Table 2, four biflavones isolated from GBL potently inhibit UGT1A1-catalyzed NHPN*-O-*glucuronidation in HLM *via* a mixed inhibition mode, with the *K*_*i*_ values of 0.07, 0.67, 0.18 and 0.74 μM, for ginkgetin, sciadopitysin, bilobetin and isoginkgetin, respectively. These findings clearly demonstrate that the biflavones in GBL are a class of naturally occurring hUGT1A1 inhibitors, which act as mixed inhibitors against hUGT1A1.

**TABLE 2:** The inhibition parameters and inhibition modes of bilobetin, isoginkgetin, sciadopitysin, ginkgetin, Amentoflavone and GBL extract against hUGT1A1-catalyzed NHPN-*O*-glucuronidation in HLM.

Inhibitor	Enzyme source	IC_50_ (μM)	*K*_*i*_ (μM)	Inhibition mode	Quality of fit (*R* ^2^)
Ginkgetin	HLM	0.15 ± 0.014 μM	0.07	Mixed	0.99
Sciadopitysin	HLM	0.28 ± 0.036 μM	0.67	Mixed	0.98
Bilobetin	HLM	0.20 ± 0.023 μM	0.18	Mixed	0.99
Isoginkgetin	HLM	0.67 ± 0.085 μM	0.74	Mixed	0.98
Amentoflavone	HLM	0.41 ± 0.034 μM	—	—	—
GBL extract	HLM	16.91 ± 1.599 μg/ml	—	—	—

### Docking Simulations

**FIGURE 6:**
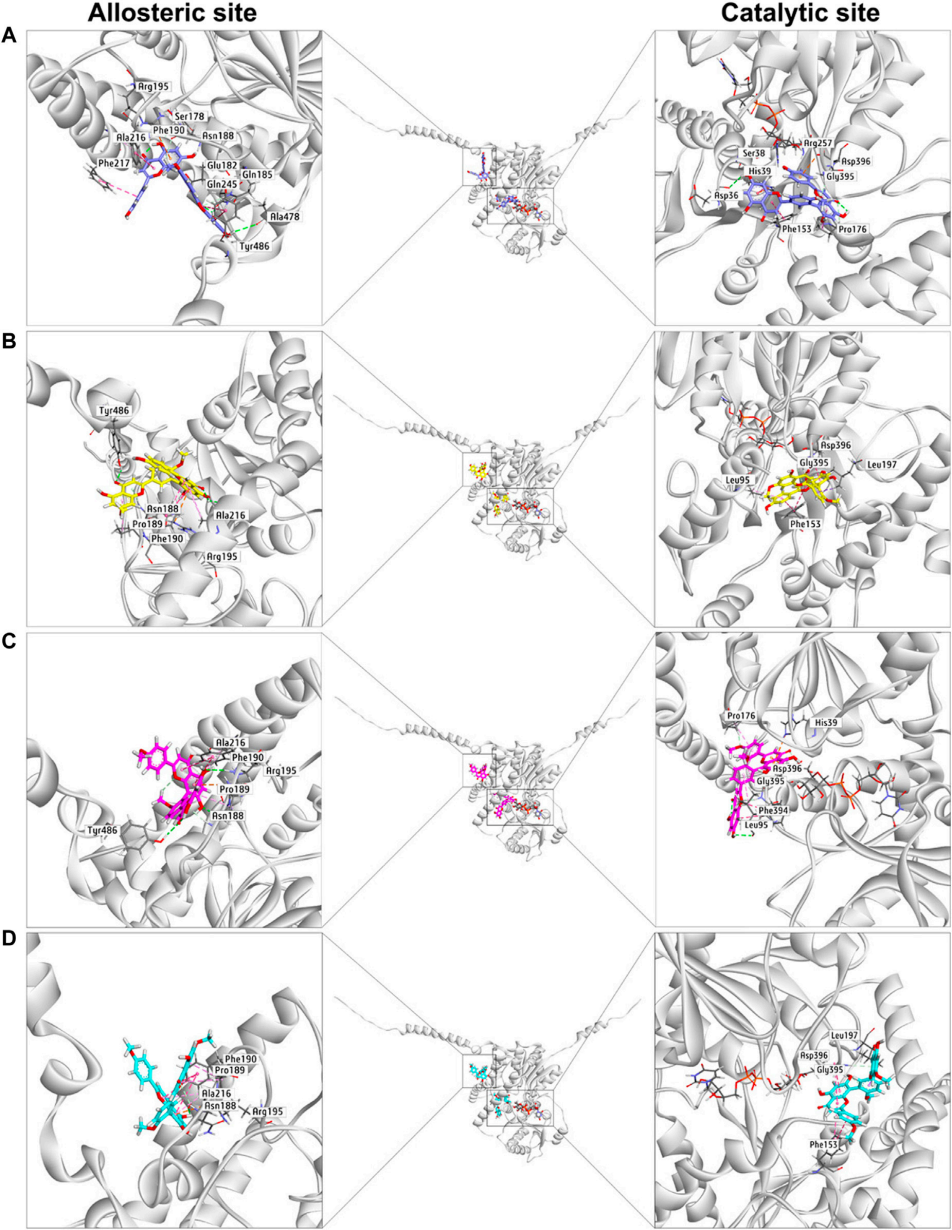
The docking simulations of four biflavones **(A)**, bilobetin in purple, **(B)**, ginkgetin in yellow, **(C)**, isoginkgetin in magenta, **(D)**, sciadopitysin in cyan) in hUGT1A1 complexed with the sugar donor UDPGA (black).

Next, docking simulations were conducted to disclose the potential binding modes of NHPN and four biflavones on hUGT1A1. As depicted in Supplementary Figure S8, NHPN and UDPGA could simultaneously bind to the catalytic pocket of hUGT1A1 (Ciotti and Owens, 1996; Locuson and Tracy, 2007). The distance between the C-4 phenolic group of NHPN and the glycosyl group of UDPGA is 2.7 Å, suggesting that NHPN is a good substrate for hUGT1A1. As depicted in Supplementary Figure S10, four biflavones also can be well-fitted into the catalytic pocket of hUGT1A1, while their binding areas are highly overlapped with that of NHPN. 2D interaction analysis showed that bilobetin, ginkgetin, sciadopitysin and NHPN created strong hydrophobic interactions with Phe153 and Leu197 in the catalytic pocket of hUGT1A1 (Supplementary Figures S6, S8, S11, S12, S14, and S15). In addition, bilobetin, isoginkgetin and NHPN bound the same key residues including His39 and Asp396 *via* hydrophobic interactions (Supplementary Figures S6, S8, S11, S13, and S15). These four bioflavones were also found to dock into the allosteric pocket of hUGT1A1, which is adjacent to its transmembrane helix (Figure 6). 2D interaction analysis showed, that these four bioflavones formed strong hydrophobic interactions with the key residues surrounding the allosteric pocket including Tyr486, Ala216, Phe190, Arg195 and Pro189 (Supplementary Figures S11, S12, S13, and S14). These observations suggest that bilobetin, ginkgetin, sciadopitysin and isoginkgetin can tightly bind on hUGT1A1 at two distinct ligand-binding sites, which well-explained the mixed-inhibition mode of these newly identified hUGT1A1 inhibitors.

## Discussion

As one of the most important conjugative enzymes in the human body, hUGT1A1 plays a crucial role in the metabolic detoxification of endogenous toxicants (e.g., bilirubin) and a variety of clinical drugs (e.g., SN38) (Rowland et al., 2013). Inhibition of hUGT1A1 by xenobiotics may trigger both, metabolic disorders of endogenous substances and clinically relevant drug/herb-drug interactions. In the present study, to overcome the high false positive rate in the *in vitro* assays of hUGT1A1 inhibition, a high-throughput cell-based fluorescence assay was established and applied in hUGT1A1 inhibitors screening from commonly used herbal medicines. Our results demonstrated that GBL extract showed the most potent hUGT1A1 inhibition in Hela-UGT1A1 cells following screening 127 herbal products, and four biflavones including bilobetin, isoginkgetin, sciadopitysin and ginkgetin, were characterized as the key constituents responsible for hUGT1A1 inhibition of GBL in living cells. These four biflavones, which were cell-permeable and are active in living cells indicated by comparable IC_50_ values of hUGT1A1 inhibition between HeLa-UGT1A1 cells and HLM, were further revealed to inhibit hUGT1A1-catalyzed NHPN-*O*-glucuronidation through mixed-inhibition modes, which was consistent with the observations that these four compounds tightly bound on hUGT1A1 in both the catalytic pocket and an allosteric pocket of hUGT1A1 in docking simulations.

*Ginkgo biloba* is among the most widely consumed herbal supplements and phytopharmaceutical drugs used in the world. The primary active constituents of GBL are flavonoids and terpene lactones, which account for 26.8 and 9.8% of the tested GBL in the present study, respectively (Supplementary materials). Our current results showed that all 12 tested flavonoids except myricetin exerted potent inhibitory activities towards NHPN-*O*-glucuronidation with IC_50_ values less than 5 μM, while the four biflavones in GBL were found to be the strongest inhibitors of hUGT1A1 with IC_50_ values ranging from 0.075 to 0.41 μM, which are 10-fold lower than those for the flavones or flavanols (1.38–3.29 μM). It is worthy to be noted that, amentoflavone, also baring a biflavone structure, showed a relatively weak inhibition on hUGT1A1, with the IC_50_ value more than 10-fold higher than those of the other four biflavones. Although it only differs from the others in the number of the methoxy groups, a lower logP value (3.49), which indicates a poorer permeability compared to the other four with logP values of 5.23–5.38, could serve as an explanation for its decreased inhibition potency on hUGT1A1 observed in Hela-UGT1A1 cells. The much lower IC_50_ value in HLM (0.41 μM) than that in Hela-UGT1A1 cells (3.29 μM) for amentoflavone may further support the poor permeability of this compound (Supplementary Figure S5 and Table 2). However, the sum effect of cell permeability of the other constituents in GBL may not be that significant, as quite similar IC_50_ values of GBL have been observed in HLM and in Hela-UGT1A1 cells (16.91 μg/ml vs 12.41 μg/ml) (Supplementary Figure S5 and Table 2).

Although there is no available data in consideration of the maximal plasma concentration (*C*_max_) of the specific four biflavones which were found to be the most potent hUGT1A1 inhibitors in the present study, the *K*_*i*_ values for these four compounds ranging from 0.15 to 0.67 μM were much higher than the measured plasma *C*_max_ of their bioflavone analogs orally administrated in rats (Chen et al., 2018). In addition, only trace amounts in the range of p.p.m. of biflavones have been determined in the used GBL extract (Supplementary Figure S4 and Supplementary Table S3), therefore, GBL is unlikely to cause a clinically significant metabolic drug/herb-drug interaction *via* bioflavone mediated inhibition of hUGT1A1 involved in drug metabolism *in vivo*. It is also worth to be noted that the flavonoids are present as glycosides but not the free aglycones in GBL, and the hUGT1A1 inhibition of aglycones tested in the present study may not been straightforward for assessing DDI-potential at the gastrointestinal site *in vivo*. In addition, a previous study showed that *ginkgo biloba* leaf extract only slightly changed the pharmacokinetics of raltegravir (mainly eliminated through UGT1A1-mediated metabolism) in healthy volunteers (Blonk et al., 2012), indicating that the pharmacokinetic interactions between *ginkgo biloba* leaf extract and UGT1A1 substrates may be minor or negligible. Moreover, bioactive constitutes from *Ginkgo biloba* leaf extract have been reported to be able to induce the expression of hepatic drug-metabolizing enzymes including UGT1A1 (Li et al., 2008). Therefore, the real influence of GBL on the pharmacokinetics of UGT1A1 drug substrates in human warrants further investigation. On the other hand, for herbal medicines co-administrated with ginkgo preparations, which undergo extensive glucuronidation metabolism mediated by intestinal hUGT1A1, higher systemic exposure is expected through the inhibition of the intestinal UGT1A1-mediated glucuronidation by chemical constituents in ginkgo preparations. Furthermore, the four bioflavones with potent hUGT1A1 inhibitory activities contained in GBL could hold the potentials to be developed as hUGT1A1 inhibitors to exhibit broad-spectrum properties through mediating a yet undefined potential drug/herb-drug interaction.

The docking simulations showed that the substrate-binding site of hUGT1A1 could be split into two embossed subdomains (Supplementary Figure S7). As shown in Supplementary Figure S7, the upper domain (UD) consists of a hydrophobic area (Pro176, Phe153, His39) and a H-bond acceptor from Asp36. The downward domain (DD) consists of a larger hydrophobic area (Leu197, Asp396, Gly395, Phe394, Leu95) and also Leu95 rendering a H-bond receptor from its back bone. Remarkably, the amide bond between Asp396 and Gly395 was greatly exposed to the surface of the substrate-binding site, which enabled an Amide-Pi stacked interaction with aromatic ring. More conspicuously, His39 and Asp396 seemed to be of great significance for the binding of flavoniod-like structures by forming electrostatic interactions. In summary, aromatic groups are highly preferred in the substrate-binding site of hUGT1A1. The binding poses of sciadopitysin and ginkgetin were highly overlapped, indicating that there is no difference in binding formation between methyl and methoxyl substitutions on R_2_ (Supplementary Figure S10). Meanwhile, isoginkgetin, sciadopitysin and ginkgetin bonded to the substrate-binding site in a similar mode with R_2_-side flavoniod structure binding to UD and R_1_-side chromone group binding to DD (Supplementary Figure S9). The R_2_-side flavoniod of isoginkgetin bonded to UD conversely as sciadopitysin and ginkgetin did to form a H-bond with Leu95, resulting in the tightest binding pose of isoginkgetin upon the substrate-binding site (Figure 6). Bilobetin, lacking of methyl on R_1_ and R_2_, bonded deeply inside into the polar internal cavity and formed a H-bond with Asp36 (Figure 6), which further immobilized bilobetin to the substrate-binding site. Furthermore, all four biflavones present in GBL, bilobetin, ginkgetin, sciadopitysin and isoginkgetin, occupied the overlapped binding area in the catalytic pocket of hUGT1A1 with NHPN, and also well fit into the allosteric pocket of hUGT1A1, by which the mixed inhibition modes of the four biflavones on NHPN-*O*-glucuronidation could be well explained (Figure 6, Supplementary Figures S10 and S11). Our previous study demonstrated that the ligand-binding site of NHPN on hUGT1A1was identical to that of bilirubin (Liu et al., 2019), thus, mixed inhibition types could be predicted in the condition where bilirubin is used as the substrate to evaluate the inhibitory effect of these biflavoneson hUGT1A1. However, how NHPN compares to the drug substrates of UGT1A1 such as belinostat or estrogens warrants further study.

## Conclusion

In summary, with the help of a newly designed high-throughput cell-based fluorescence assay, the inhibitory potentials of a number of commonly used herbal medicines against hUGT1A1 were assayed in living cells. The results demonstrated that GBL extract used in the present study displayed the most potent hUGT1A1 inhibition potency, with the inhibition rate exceeding 95% at the dose of 100 μg/ml. Further investigations showed that four biflavones including bilobetin, isoginkgetin, sciadopitysin and ginkgetin, are key constituents responsible for hUGT1A1 inhibition in living cells, while the flavonoids in GBL contribute to a lesser extent for hUGT1A1 inhibition. Inhibition kinetic analyses suggested that the biflavones in GBL potently inhibit hUGT1A1-catalyzed NHPN-*O*-glucuronidation in HLM *via* a mixed-inhibition manner, with the *K*_*i*_ values ranging from 0.07 to 0.74 μM. Docking simulations suggested that bilobetin, ginkgetin, sciadopitysin and isoginkgetin could tightly bind on hUGT1A1 at two distinct ligand-binding sites (one is located at the catalytic pocket of hUGT1A1 and another one is an allosteric pocket which was adjacent to the transmembrane helix), which may be a plausible explanation for the mixed-inhibition mode of these newly identified hUGT1A1 inhibitors. In conclusion, our findings revealed that the biflavones in GBL are potent hUGT1A1 inhibitors, which can be very helpful for guiding the rational use of GBL-related herbal products in clinical settings.

## Data Availability

The original contributions presented in the study are included in the article/Supplementary Material, further inquiries can be directed to the corresponding authors.
