# Supplementary material for: Discovery and Characterization of the Key Constituents in Ginkgo biloba Leaf Extract With Potent Inhibitory Effects on Human UDP-Glucuronosyltransferase 1A1
Source: Front Pharmacol. 2022 Feb 21;13:815235. doi: 10.3389/fphar.2022.815235 (PMC8899474; doi:10.3389/fphar.2022.815235)
Supplement: Supplementary file 1 [file DataSheet1.docx]

# Supplementary materials

*for*

# Discovery and characterization of the key constituents in *Ginkgo biloba* leaf extract with potent inhibitory effects on human UDP-glucuronosyltransferase 1A1

**Hui-Lin Pang^1, †^, Guang-Hao Zhu^2,^ ^†^, Qi-Hang Zhou^2^, Chun-Zhi Ai^4^, Ya-Di Zhu^2^, Ping Wang^2^, Tong-Yi Dou^1^, Yang-Liu Xia^1,*^, Hong Ma^3,*^, Guang-Bo Ge^2,*^**

^1^School of Life and Pharmaceutical Sciences, Dalian University of Technology, Panjin 124221, China.

^2^Shanghai Frontiers Science Center for Chinese Medicine Chemical Biology, Institute of Interdisciplinary Integrative Medicine Research, Shanghai University of Traditional Chinese Medicine, Shanghai 201203, China.

^3^Shanghai Research Institute of Acupuncture and Meridian, Shanghai University of Traditional Chinese Medicine, Shanghai 200030, China.

^4^State Key Laboratory for Chemistry and Molecular Engineering of Medicinal Resources, School of Chemistry and Pharmacy, Guangxi Normal University, 15 Yucai Road, Guilin 541004, China.

^†^These authors contributed equally to this work and share first authorship

*** Correspondence**

Guang-Bo Ge

geguangbo@dicp.ac.cn

Hong Ma

redlemononly@iCloud.com

Yang-Liu Xia

xiayl@dlut.edu.cn


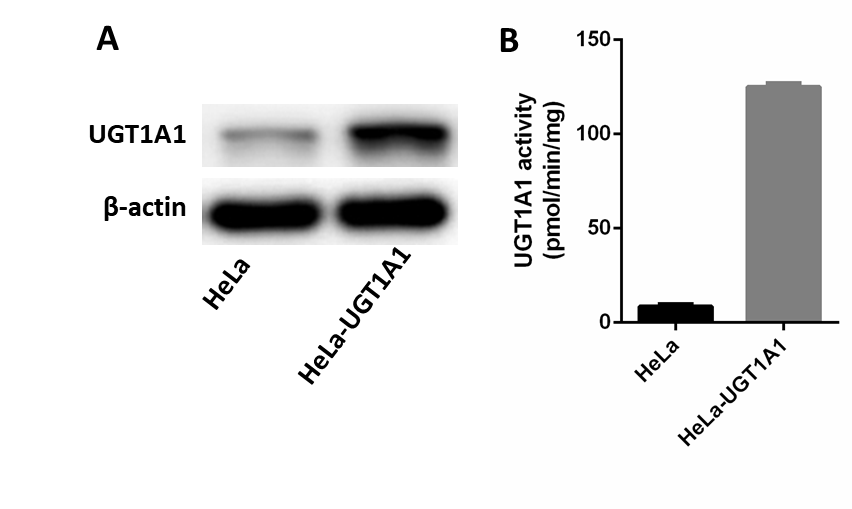


**Fig. S1.** The validation of UGT1A1 protein expression and activity in HeLa-UGT1A1 cells by using Western blotting and probe reaction.

**Table S1.** Inhibitory effects of herbal medicines (10 μg/mL) on UGT1A1-catalyzed NHPN-*O*-glucuronidation in HeLa-UGT1A1 cells. Each data expressed as the mean of triplicate assays.

| **No.** | **Herbal medicines** | **Residual activity (%)** | **No.** | **Herbal medicines** | **Residual activity (%)** |
| --- | --- | --- | --- | --- | --- |
| 1 | *Finger Citron* **(Fruit)** | 143.8 | 64 | *Gordon Euryale* **(Seed)** | 96.54 |
| 2 | *Stichy Rice* **(Root)** | 132.2 | 65 | *Ginger* **(Fresh Rhizome)** | 96.5 |
| 3 | *Solomonseal* **(Rhizome)** | 131.3 | 66 | *Ophicalcite* | 96.44 |
| 4 | *Senna* **(Leaf)** | 126.1 | 67 | *Tangerine* **(Seed)** | 96.3 |
| 5 | Bitter Apricot **(Peeled Seed)** | 121.5 | 68 | *Bletilla* **(Rhizome)** | 96.19 |
| 6 | *Ginger Processed Pinellia* **(Tuber)** | 120.7 | 69 | *Chrysan-themum* **(Flower)** | 95.55 |
| 7 | Milkvetch **(Root)** | 120.6 | 70 | *Turmeric* **(Longitudinal Section)** | 95.33 |
| 8 | Oriental Waterplantain **(Tuber)** | 120.1 | 71 | *Yam* **(Common Rhizome)** | 95.08 |
| 9 | *Herba Centipedae* **(Herb)** | 118.9 | 72 | *Mulberry* **(Branch)** | 94.63 |
| 10 | *Glabrous Greenbrier* **(Rhiaome)** | 119.5 | 73 | *Cassia* **(Twig)** | 94.07 |
| 11 | *Faeces Trogopterpri*  **(Processed with vinegar)** | 118.8 | 74 | *Haematitum* **(Calcined)** | 94 |
| 12 | Barbary Wolfberry **(Fruit)** | 118.2 | 75 | *Tangerine***(Green Peel Processed with Vinegar)** | 93.63 |
| 13 | *Schisandra*  **(Seed Processed with vinegar)** | 117.9 | 76 | *Rehmannia*  **(Root Processed with Wine)** | 93.24 |
| 14 | *Lychee* **(Seed)** | 117.2 | 77 | *Caulis Spatholobi* **(Cane)** | 92.89 |
| 15 | *Himalayan Stachyurus* **(Pith)** | 116.6 | 78 | *Lotus* **(Seed)** | 92.17 |
| 16 | *Officinal Magnolia* **(Flower)** | 116.4 | 79 | *Indigo Naturalis* **(Leaf )** | 91.84 |
| 17 | Nutmeg | 115.4 | 80 | *Pogodatree***(Stir-fried Flower)** | 90.85 |
| 18 | *Tangerine* **(Red Exocarpium)** | 115.2 | 81 | *Clam Shell* | 90.71 |
| 19 | *Puffball* **(Thallus)** | 115.1 | 82 | *Coix* **(Seed)** | 90.66 |
| 20 | *Mulberry* **(Fruit)** | 114.7 | 83 | *Cyrtomium Fortunei***(Rhizome)** | 89.86 |
| 21 | *Licorice* **(Baked Root)** | 114.5 | 84 | *Hyacinth Dolichos* **(Flower)** | 89.64 |
| 22 | *Chinese Angelica* **(Root)** | 113.6 | 85 | *Feculae Bombycis* | 89.49 |
| 23 | *Ampelopsis Japonica* **(Root)** | 111.9 | 86 | *Field Thistle* **(Herb)** | 88.65 |
| 24 | *Dayflower* **(Herb)** | 111.8 | 87 | *Lotus* **(Leaf)** | 88.36 |
| 25 | *Clove* **(Flower)** | 111.6 | 88 | *Mahonia* **(Leaf)** | 88.26 |
| 26 | *Goldthread* **(Rhizome)** | 110.5 | 89 | *Weeping Forsythia* **(Fruit)** | 87.73 |
| 27 | *Ginseng*  **(Red Root and Rhizome)** | 110.3 | 90 | *Indian Mockstrawberry* **(Herb)** | 87.35 |
| 28 | *Cardamon* **(Fruit)** | 110.3 | 91 | *Reed* **(Rhizome)** | 86.9 |
| 29 | *Ginkgo* **(Seed)** | 110 | 92 | *Pumex* | 86.64 |
| 30 | *Capejasmine* **(Fruit)** | 110 | 93 | *Notopterygium***(Rhizome and Root)** | 85.82 |
| 31 | *Buckeye* **(Seed)** | 110 | 94 | *Pyrola* **(Herb)** | 85.1 |
| 32 | *Ginger* **(Dried Rhizome)** | 109.1 | 95 | *Chinese Arborvitae***(Twig and Leaf)** | 84.58 |
| 33 | *Snakegourd* **(Fruit)** | 108.1 | 96 | *Epimedium* **(Herb)** | 84.29 |
| 34 | *Plantain* **(Herb)** | 107.9 | 97 | *Hirsute Bugleweed* **(Herb)** | 84.02 |
| 35 | *Angelica* **(Root)** | 107 | 98 | *Millet Sprout* **(Fried)** | 82.36 |
| **No.** | **Herbal medicines** | **Residual activity (%)** | **No.** | **Herbal medicines** | **Residual activity (%)** |
| 36 | *Pricklyash* **(Pericarp)** | 106.7 | 99 | *Cablin Potchouli* **(Herb)** | 82.07 |
| 37 | *Hogfennel* **(Root)** | 106.3 | 100 | *Asiatic Cornelian Cherry*  **(Dried Fruit)** | 81.68 |
| 38 | *Lily* **(Bulb)** | 105.4 | 101 | *Scouring Rush*  **(Common Herb)** | 81.22 |
| 39 | *Hemp* **(Fruit)** | 105.2 | 102 | *Chlorite Schist* | 80.99 |
| 40 | *Pipewort* **(Flower)** | 105 | 103 | *Chinese Pulsatilla* **(Root)** | 80.11 |
| 41 | *Climbing Fern Spore* | 104.9 | 104 | *Zedoray*  **(Rhizome Processed with Vinegar)** | 79.5 |
| 42 | *Wheat*  **(Fruit of Huai Valleys Grown)** | 104.9 | 105 | *Cassia* **(Bark)** | 79.49 |
| 43 | *Long Pepper* **(Fruit)** | 104.7 | 106 | *Drynariae* **(Rhizome)** | 79.07 |
| 44 | *Glycinemax (L.)Merr.*  **(Black Testa)** | 104.3 | 107 | Mulberry **(Leaf)** | 78.91 |
| 45 | *Cluster Mallow* **(Fruit)** | 104.3 | 108 | *Orientvine* **(Stem)** | 78.13 |
| 46 | *Cynanchum Atratum*  **(Root and Rhizome)** | 103.7 | 109 | *Grape* **(Wild Root)** | 77.26 |
| 47 | *Perilla* **(Fried Fruit)** | 103.4 | 110 | *Fleeceflower*  **(Tuber and Stem)** | 77.18 |
| 48 | Mulberry (Bark Processed with Honey) | 102.8 | 111 | *American Ginseng* **(Root)** | 76.49 |
| 49 | *Cassia* **(Fried Seed)** | 102.4 | 112 | *Glabrous Greenbrier* **(Rhiaome)** | 74.65 |
| 50 | *Malaytea Scurfpea*  (**Fruit Processed with Salt-Water)** | 102 | 113 | *Rugose Rose* **(Flower)** | 74.14 |
| 51 | *Motherwort* **(Fruit)** | 101.1 | 114 | *Kirilow Rhodiola*  **(Root and Rhizome)** | 74.09 |
| 52 | *Chinese Lobelia* **(Herb)** | 101 | 115 | *Magnolia Bark*  **(Cooked in Ginger Soup)** | 73.36 |
| 53 | *Fineleaf Schizonepeta* **(Herb)** | 100.9 | 116 | *Cuttle Bone* | 73.12 |
| 54 | *Hawthorn* **(Fruit)** | 100.2 | 117 | *Chinese Actinidia* **(Root)** | 71.56 |
| 55 | *Dark Plum* **(Fruit)** | 100 | 118 | *Platycodon* **(Root)** | 71.3 |
| 56 | *Buead with Pine* **(Root)** | 98.67 | 119 | *Bile Arisaema* | 70.39 |
| 57 | *Buffalo Horn* | 98.37 | 120 | *Cogon Grass* **(Rhizome)** | 70.12 |
| 58 | *Zingberis* **(Preparata Rhizoma)** | 98.36 | 121 | *Peppermint* | 68.29 |
| 59 | *Aloes* | 97.96 | 122 | *Combined Spicebush* **(Root)** | 67.26 |
| 60 | *Akebia Stem* | 97.5 | 123 | *Arnebia* **(Root)** | 65.06 |
| 61 | *Medicine Terminalia* **(Fruit)** | 97.41 | 124 | *Vitex Trifolia* **(Fried Fruit)** | 63.26 |
| 62 | *Tangerine* **(Dried Peel)** | 97.07 | 125 | *Barbated Skullcup* **(Herb)** | 62.43 |
| 63 | *Lobed Kudzuvine* **(Root)** | 96.67 | 126 | *Fennel*  **(Fruit Processed with Salt-Water)** | 61.93 |
| - | *-* | - | 127 | *Ginkgo* **(Leaf)** | 39.05 |

**The composition of the *Ginkgo biloba* leaf extract**

The standard *Ginkgo biloba* leaf extract (GBL) was obtained from Shanghai Sine Promod Pharmaceutical Co., Ltd (Shanghai, China). The green leaves of *Ginkgo biloba* were collected in Oct 2017 from Enshi, Hubei, China. A voucher specimen was deposited in the manufacture after authentication. Solvents used for extraction were methanol, ethanol, chloroform, hexane, and water with drug to extract ratio of 50 g/200 mL (w/v). The contents of flavonol glycosides, terpene trilactones and ginkgolic acids were 26.8%, 9.8% and 2.9 mg/kg.

The chemical analysis of *Ginkgo biloba* leaf extract was analyzed by using ultra high performance liquid Chromatography-Q exactive hybrid quadrupole orbitrap high-resolution accurate mass spectrometric (UHPLC-Q-Orbitrap HRMS, Thermo Fisher Scientific Inc., Grand Island, NY, USA). The UHPLC was Thermo Scientific Dionex Ultimate 3000 and controlled by Chromeleon 7.2 Software. The cooling autosampler was set at 10°C and protected from light, and the column heater was set at 40°C. A Waters ACQUITY UPLC BEH C_18_ column (2.1 × 100 mm, 1.7 μm) was employed. The mobile phase consisted of A (methanol) and B (0.1% formic acid) at a flow rate of 0.3 mL·min^−1^ and eluted with gradient elution: 0-4 min (4% A), 4-10 min (4%-12% A), 10-30 min (12%-70% A), 30-35 min (70% A), 35-38 min (70%-95% A), 38-42 min (95% A), 42-45 min (95%-4% A). The injection volume was 2 μL.

The mass spectrometer Q-Orbitrap system was connected to the UHPLC system via a heated electrospray ionization and controlled by Xcalibur 4.1 software which was used for data capture and analysis. The electrospray ionization source was operated and optimized in negative ionization model. The optimized parameters of mass spectrometry were as follows: capillary temperature: 320°C; sheath gas (N_2_) flow rate: 35 arbitrary units; auxiliary gas (N_2_) flow rate: 13 arbitrary units; sweep gas flow rate: 0 arbitrary units; spray voltage: 2.5 kV; S-lens RF level: 50V; auxiliary gas heater temperature: 300°C; scan mode: full MS; scan range: 80-1200 *m/z*; maximum injection time (IT): 200 ms; scan resolution: 70,000 FWHM (m/z/s); automatic gain control (AGC) target: 1.0e^6^. The protonated molecular weights of all identified compounds were calculated within an error of 10 ppm. The typical chromatographic fingerprint of Ginkgo Leaf extract was deposited in **Fig.S2**. By comparing the retention times and MS/MS spectra with those of the reference standards, a total of 51 chemicals were identified or tentatively characterized from *Ginkgo biloba* leaf extract (**Table S2**), including 32 flavonol glycosides, 5 terpene trilactones, 5 biflavones, 3 flavanols, 4 flavonols, and 2 organic acids.


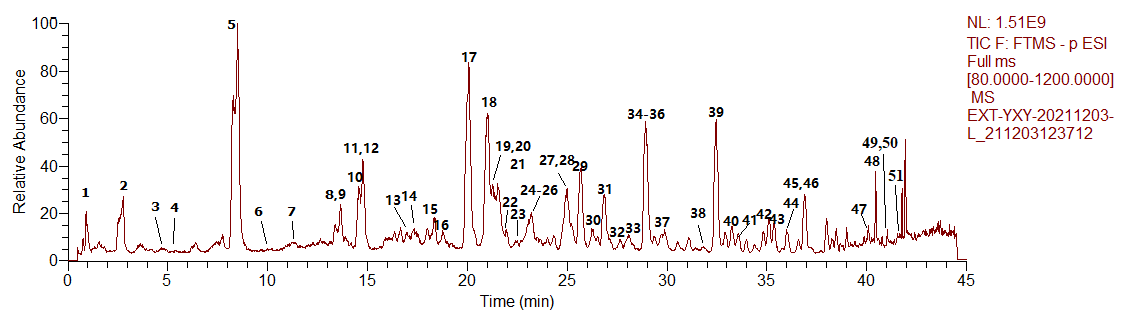


**Fig. S2** The total ion chromatograms (TICs) of *Ginkgo biloba* leaf extract in negative mode by UHPLC-Q-Exactive Orbitrap HRMS

**Table S2** Characterization of chemical constituents of *Ginkgo biloba* leaf extract by UHPLC-Q-Exactive Orbitrap HRMS

| **No.** | **RT**  **(min)** | **Ion mode** | **Measured mass/Da** | **Calculated mass/Da** | **Error/ppm** | **Molecular formula** | **Identification** | **Type** |
| --- | --- | --- | --- | --- | --- | --- | --- | --- |
| 1 | 0.89 | [M-H]^-^ | 191.05536 | 191.05501 | 1.808 | C7H12O6 | Quinic Acid | organic acids |
| 2 | 2.69 | [M-H]^-^ | 153.01814 | 153.01824 | 0.622 | C7H6O4 | protocatechuic acid | organic acids |
| 3 | 4.78 | [M-H]^-^ | 305.06699 | 305.06558 | 4.625 | C15H14O7 | gallocatechin | flavanols |
| 4 | 5.18 | [M-H]^-^ | 289.07202 | 289.07066 | 4.689 | C15H14O6 | Catechin | flavanols |
| 5 | 8.51 | [M-H]^-^ | 325.09302 | 325.09179 | 3.772 | C15H18O8 | Bilobalide | terpene trilactones |
| 6 | 10.03 | [M-H]^-^ | 289.07205 | 289.07066 | 4.793 | C15H14O6 | Epicatechin | flavanols |
| 7 | 11.4 | [M-H]^-^ | 771.20007 | 771.19783 | 2.899 | C33H40O21 | 3-O-[β-d-D-glucopyranose (1"→2")]-[α-L-rhamnopyranose (1"→6")]-β-d-glucopyranosyl quercetin | flavonol glycosides |
| 9 | 13.66 | [M-H]^-^ | 423.13019 | 423.12857 | 3.821 | C20H24O10 | Ginkgolide J | terpene trilactones |
| 10 | 14.44 | [M-H]^-^ | 771.2002 | 771.19783 | 3.067 | C33H40O21 | 3-O-[β-d-D-glucopyranose (1"→2")]-[α-L-rhamnopyranose (1"→6")]-β-d-glucopyranosyl quercetin tautomer | flavonol glycosides |
| 11 | 14.63 | [M-H]^-^ | 755.20435 | 755.20292 | 1.894 | C33H40O20 | 3-O-[β-d-D-glucopyranose (1"→2")]-[α-L-rhamnopyranose (1"→6")]-β-d-glucopyranosyl kaempferol | flavonol glycosides |
| 12 | 14.79 | [M-H]^-^ | 439.12479 | 439.12349 | 2.965 | C20H24O11 | Ginkgolide C | terpene trilactones |
| 13 | 16.97 | [M-H]^-^ | 625.14166 | 625.13993 | 2.774 | C27H30O17 | 3-O-[α-L-rhamnopyranose (1"→6")]-β-d-glucopyranosyl poppyflavone | flavonol glycosides |
| 14 | 17.33 | [M-H]^-^ | 755.2052 | 755.20292 | 3.019 | C33H40O20 | 3-O-[β-d-D-glucopyranose (1"→2")]-[α-L-rhamnopyranose (1"→6")]-β-d-glucopyranosyl kaempferol tautomer | flavonol glycosides |
| 15 | 18.36 | [M-H]^-^ | 755.20477 | 755.20292 | 2.45 | C33H40O20 | 3-O-[α-L-rhamnopyranose (1"→2")]-[α-L-rhamnopyranose (1"→6")]-β-d-glucopyranosyl quercetin | flavonol glycosides |
| 16 | 18.81 | [M-H]^-^ | 287.05637 | 287.05501 | 4.722 | C15H12O6 | Dihydrokaempferol | flavonol glycosides |
| 17 | 20.05 | [M-H]^-^ | 407.13492 | 407.13366 | 3.098 | C20H24O9 | Ginkgolide A | terpene trilactones |
| 18 | 21.02 | [M-H]^-^ | 423.12994 | 423.12857 | 3.23 | C20H24O10 | Ginkgolide B | terpene trilactones |
| 19 | 21.27 | [M-H]^-^ | 463.0885 | 463.08710 | 3.018 | C21H20O12 | 3-O-β-d-glucopyranosyl quercetin | flavonol glycosides |
| 20 | 21.27 | [M-H]^-^ | 739.21008 | 739.20801 | 2.807 | C33H40O19 | 3-O-[α-L-rhamnopyranose (1"→2")]-[α-L-rhamnopyranose (1"→6")]-β-d-glucopyranosyl kaempferol | flavonol glycosides |
| 21 | 21.52 | [M-H]^-^ | 609.14661 | 609.14501 | 2.625 | C27H30O16 | Rutinum | flavonol glycosides |
| 22 | 21.96 | [M-H]^-^ | 769.22083 | 769.21857 | 2.938 | C34H42O20 | 3-O-[α-L-rhamnopyranose (1"→2")]-[α-L-rhamnopyranose (1"→6")]-β-d-glucopyranosyl isorhamnetin | flavonol glycosides |
| 23 | 22.19 | [M-H]^-^ | 639.15723 | 639.15558 | 2.588 | C28H32O17 | 3-O-[β-d-D-glucopyranosyl (1"→2")]-α-L-rhamnopyranose-3'-methylparaben | flavonol glycosides |
| 24 | 22.98 | [M-H]^-^ | 901.24207 | 901.23970 | 2.631 | C42H46O22 | 3-O-{6-O-[β-d-D-glucopyranosyl (1"→6")]-coumaroyl-β-d-D-glucopyranosyl (1"→2")}-α-L-rhamnopyranose-kaempferol | flavonol glycosides |
| 25 | 23.15 | [M-H]^-^ | 447.09363 | 447.09219 | 3.226 | C21H20O11 | 3-O-β-d-glucopyranosyl kaempferol isomers | flavonol glycosides |
| 26 | 23.19 | [M-H]^-^ | 639.15723 | 639.15558 | 2.588 | C28H32O17 | 3-O-[α-L-rhamnopyranose (1"→6")]-β-d-glucopyranose-3'-methyl poplarin | flavonol glycosides |
| 27 | 24.99 | [M-H]^-^ | 609.14673 | 609.14501 | 2.822 | C27H30O16 | 3-O-[β-d-D-glucopyranosyl (1"→2")]-α-L-rhamnopyranose-quercetin | flavonol glycosides |
| **No.** | **RT**  **(min)** | **Ion mode** | **Measured mass/Da** | **Calculated mass/Da** | **Error/ppm** | **Molecular formula** | **Identification** | **Type** |
| 28 | 25.23 | [M-H]^-^ | 447.09366 | 447.09219 | 3.293 | C21H20O11 | 3-O-β-d-glucopyranosyl kaempferol | flavonol glycosides |
| 29 | 25.69 | [M-H]^-^ | 593.15161 | 593.15010 | 2.552 | C27H30O15 | 3-O-[α-L-rhamnopyranose (1"→6")]-β-d-glucopyranosyl kaempferol | flavonol glycosides |
| 30 | 26.3 | [M-H]^-^ | 477.10406 | 477.10275 | 2.741 | C22H22O12 | 3-O-β-d-glucopyranosyl isorhamnetin | flavonol glycosides |
| 31 | 26.86 | [M-H]^-^ | 623.16235 | 623.16066 | 2.71 | C28H32O16 | 3-O-[β-d-D-glucopyranosyl (1"→2")]-α-L-rhamnopyranose-isorhamnetin | flavonol glycosides |
| 32 | 27.69 | [M-H]^-^ | 653.17328 | 653.17123 | 3.145 | C29H34O17 | 3-O-[α-L-rhamnopyranose (1"→6")]-β-d-glucopyranosylbutyranoside | flavonol glycosides |
| 33 | 28.06 | [M-H]^-^ | 447.09369 | 447.09219 | 3.36 | C21H20O11 | 3'-O-β-D-glucopyranosyl lignocerebrosidin | flavonol glycosides |
| 34 | 28.9 | [M-H]^-^ | 593.151 | 593.15010 | 1.523 | C27H30O15 | 3-O-[β-d-D-glucopyranosyl (1"→2")]-α-L-rhamnopyranose-kaempferol | flavonol glycosides |
| 35 | 28.9 | [M-H]^-^ | 755.18341 | 755.18179 | 2.145 | C36H36O18 | 3-O-[6-O-Coumaroyl-β-d-D-glucopyranosyl (1"→2")]-α-L-rhamnopyranose-quercetin | flavonol glycosides |
| 36 | 29.09 | [M-H]^-^ | 653.17297 | 653.17123 | 2.67 | C29H34O17 | 3-O-[β-d-D-glucopyranosyl (1"→2")]-α-L-rhamnopyranose-butyranoside | flavonol glycosides |
| 37 | 29.71 | [M-H]^-^ | 301.03546 | 301.03428 | 3.923 | C15H10O7 | Quercetin | flavonol |
| 38 | 31.76 | [M-H]^-^ | 285.04065 | 285.03936 | 4.51 | C15H10O6 | Luteolin | flavonol |
| 39 | 32.46 | [M-H]^-^ | 739.18884 | 739.18688 | 2.657 | C36H36O17 | 3-O-[6-O-Coumaroyl-β-d-D-glucopyranosyl (1"→2")]-α-L-rhamnopyranose-kaempferol | flavonol glycosides |
| 40 | 33.22 | [M-H]^-^ | 769.19928 | 769.19744 | 2.391 | C37H38O18 | 3-O-[6-O-Coumaroyl-β-d-D-glucopyranosyl (1"→2")]-α-L-rhamnopyranose-isorhamnetin | flavonol glycosides |
| 41 | 33.58 | [M-H]^-^ | 755.18378 | 755.18179 | 2.635 | C36H36O18 | 3-O-[6-O-Coumaroyl-β-d-D-glucopyranosyl (1"→2")]-α-L-rhamnopyranose-quercetin tautomer 2 | flavonol glycosides |
| 42 | 35.11 | [M-H]^-^ | 285.04047 | 285.03936 | 3.879 | C15H10O6 | [kaempferol](http://dict.cn/kaempferol) | flavonol glycosides |
| 43 | 35.36 | [M-H]^-^ | 739.18903 | 739.18688 | 2.914 | C36H36O17 | 3-O-[6-O-Coumaroyl-β-d-D-glucopyranosyl (1"→2")]-α-L-rhamnopyranose-kaempferol tautomer 1 | flavonol glycosides |
| 44 | 36.00 | [M-H]^-^ | 269.04562 | 269.04445 | 4.349 | C15H10O5 | Apigenin | flavonol |
| 45 | 36.84 | [M-H]^-^ | 315.05121 | 315.04993 | 4.066 | C16H12O7 | Isorhamnetin | flavonol |
| 46 | 36.90 | [M-H]^-^ | 739.18823 | 739.18688 | 1.832 | C36H36O17 | 3-O-[6-O-Coumaroyl-β-d-D-glucopyranosyl (1"→2")]-α-L-rhamnopyranose-kaempferol tautomer 2 | flavonol glycosides |
| 47 | 40.10 | [M-H]^-^ | 537.08307 | 537.08162 | 2.694 | C30H18O10 | amentoflavone | biflavones |
| 48 | 40.42 | [M-H]^-^ | 551.09869 | 551.09727 | 2.571 | C31H20O10 | bilobetin | biflavones |
| 49 | 41.14 | [M-H]^-^ | 565.11426 | 565.11292 | 2.365 | C32H22O10 | ginkgetin | biflavones |
| 50 | 41.74 | [M-H]^-^ | 579.12982 | 579.12857 | 2.153 | C33H24O10 | sciadopitysin | biflavones |

**
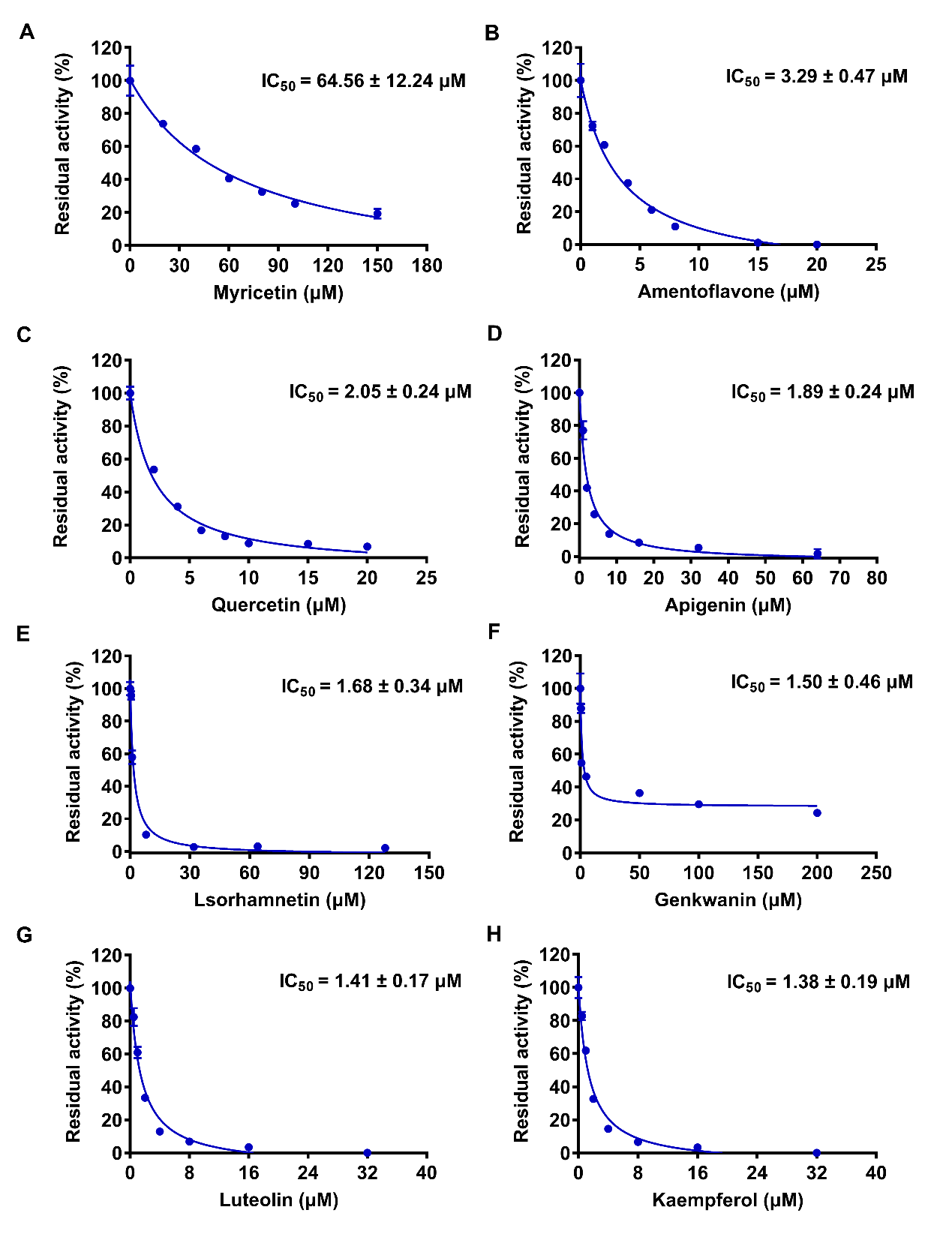
**

**Fig. S3** The dose-dependent inhibition curves of myricetin (A), amentoflavone (B), quercetin (C), apigenin (D), lsorhamnetin (E), genkwanin (F), luteolin (G), kaempferol (H) on NHPN-*O*-glucuronidation in HeLa-UGT1A1 cells. The data were expressed as the means of triplicate assays.

**Quantification of five biflavones in *Ginkgo biloba* leaf extract**

The content of five biflavones, including amentoflavone, bilobetin, ginkgetin, isoginkgetin, sciadopitysin in Ginkgo Leaf extract were determined by HPLC (Aglient 1260). The separation was conducted on a CAPCELL PAK C18 column (150 mm×4.6 mm, 5 µm) maintained at 30 °C. The mobile phase was consisted of A (acetonitrile) and B (water) at a flow rate of 1 mL/min and eluted with the following gradient: 0-20 min (30-45% A), 20-50 min (45-47% A), 50-53 min (47-80% A), 53-62 min (80% A). The injection concentration was 25.18 mg/ml and the injection volume was 10 μl. The typical chromatographic fingerprints of Ginkgo Leaf extract and the mixed reference standards of amentoflavone, bilobetin, ginkgetin, isoginkgetin, sciadopitysin were deposited in Fig. S4. The contents of amentoflavone, bilobetin, ginkgetin, isoginkgetin and sciadopitysin in *Ginkgo biloba* leaf extract were determined as 5.89 μg/g, 12.07 μg/g, 7.06 μg/g, 12.78 μg/g and 1.20 μg/g, respectively (Table S3).


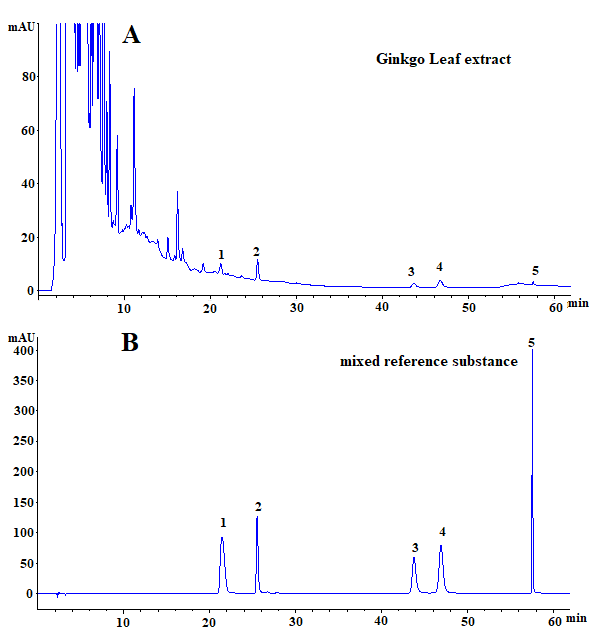


**Fig. S4** The HPLC analysis of biflavones in *Ginkgo biloba* leaf extract (A) and mixed reference standards (B) under UV detection at 340 nm. 1. amentoflavone; 2. bilobetin; 3. ginkgetin; 4. isoginkgetin; 5. sciadopitysin.

**Table S3** Quantification of five biflavones, amentoflavone, bilobetin, ginkgetin, isoginkgetin and sciadopitysin, in GBL extract using HPLC-UV

| **Tested compounds** | **Concentration of GBL extract (mg/mL)** | **Peak area of tested compounds in GBL extract** | **Concentration of reference compounds (μg/mL)** | **Peak area of reference compounds** | **Concentration of tested compounds in GBL extract (μg/mL)** | **Contents of tested compounds in GBL extract (μg/g)** |
| --- | --- | --- | --- | --- | --- | --- |
| amentoflavone | 25.18 | 65.70 | 7.37 | 3265.8 | 0.15 | 5.89 |
| bilobetin | 25.18 | 118.20 | 4.77 | 1854.1 | 0.30 | 12.07 |
| ginkgetin | 25.18 | 53.20 | 6.33 | 1896.4 | 0.18 | 7.06 |
| isoginkgetin | 25.18 | 99.10 | 9.00 | 2771.2 | 0.32 | 12.78 |
| sciadopitysin | 25.18 | 12.70 | 7.00 | 2944.4 | 0.03 | 1.20 |







(B)

(A)

**Fig. S5** The dose-dependent inhibition curves of amentoflavone (A), and GBL (B) on NHPN-*O*-glucuronidation in HLM. The data were expressed as the means of triplicate assays.


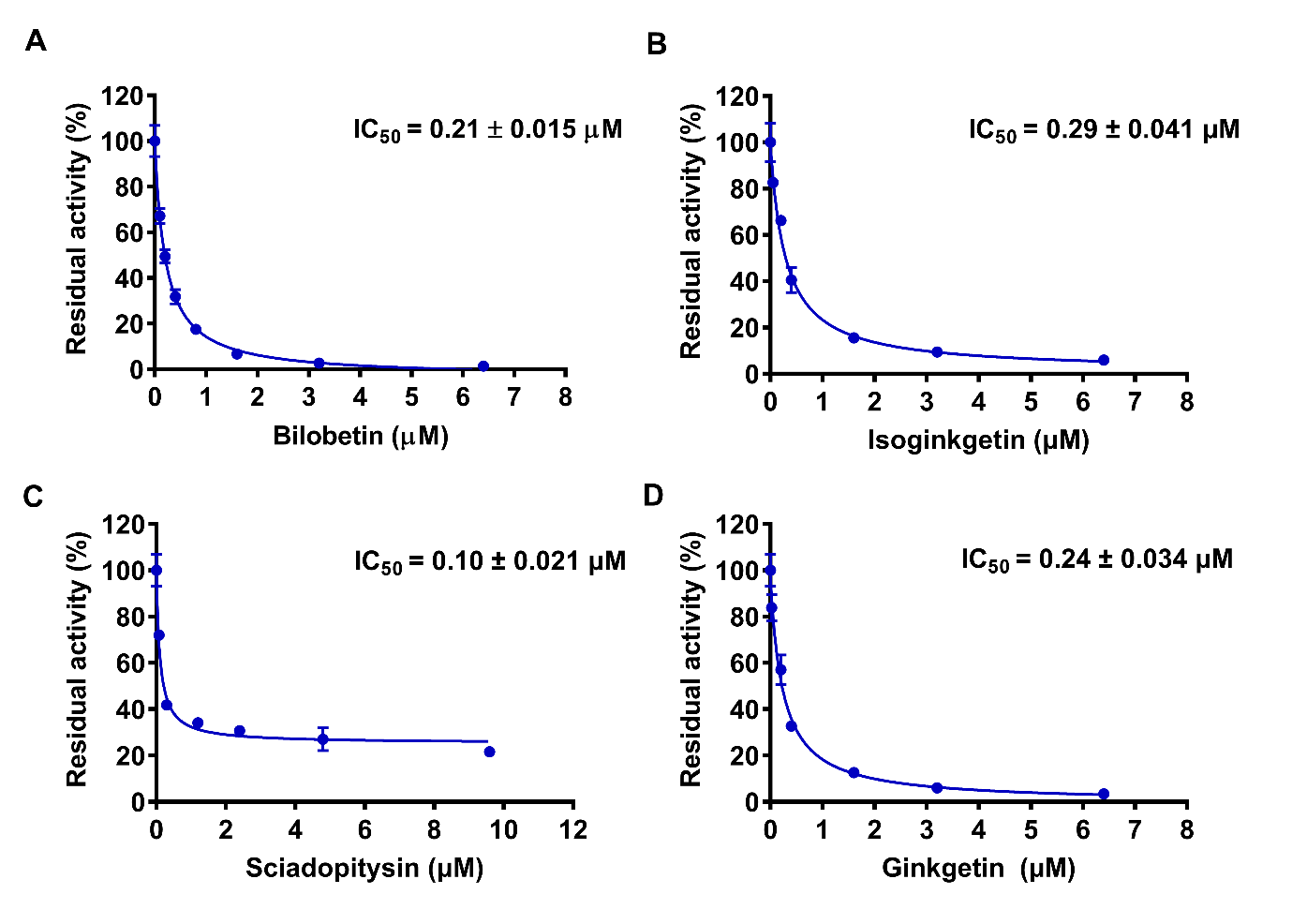


**Fig. S6** The dose-dependent inhibition curves of bilobetin (A), isoginkgetin (B), sciadopitysin (C) and ginkfetin (D) on NHPN-O-glucuronidation in recombinant UGT1A1. The data were expressed as the means of triplicate determinations.


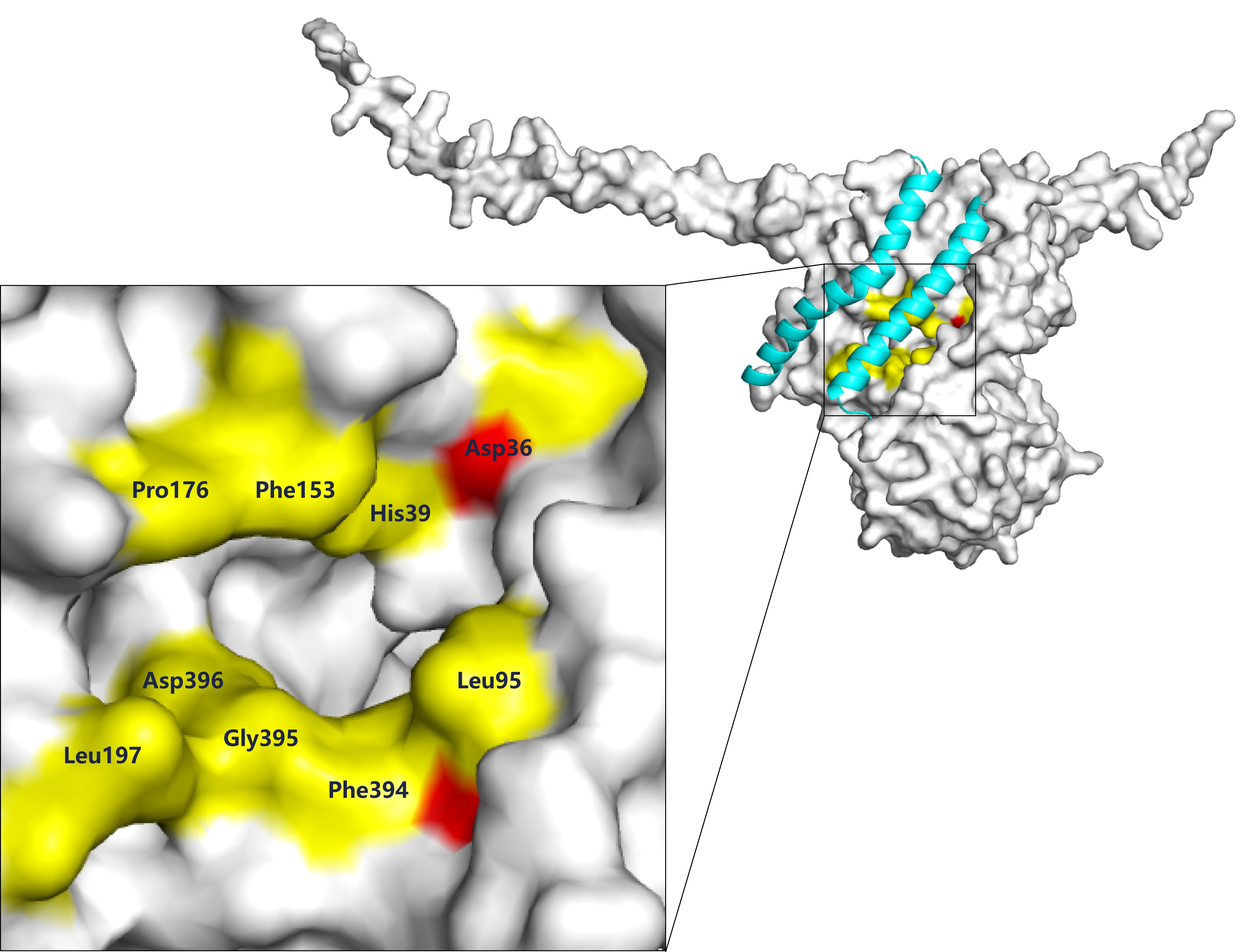


**Fig.S7** Substructure of substrate-binding site of hUGT1A1. Two superficial hydrogen-bond receptors were in red.


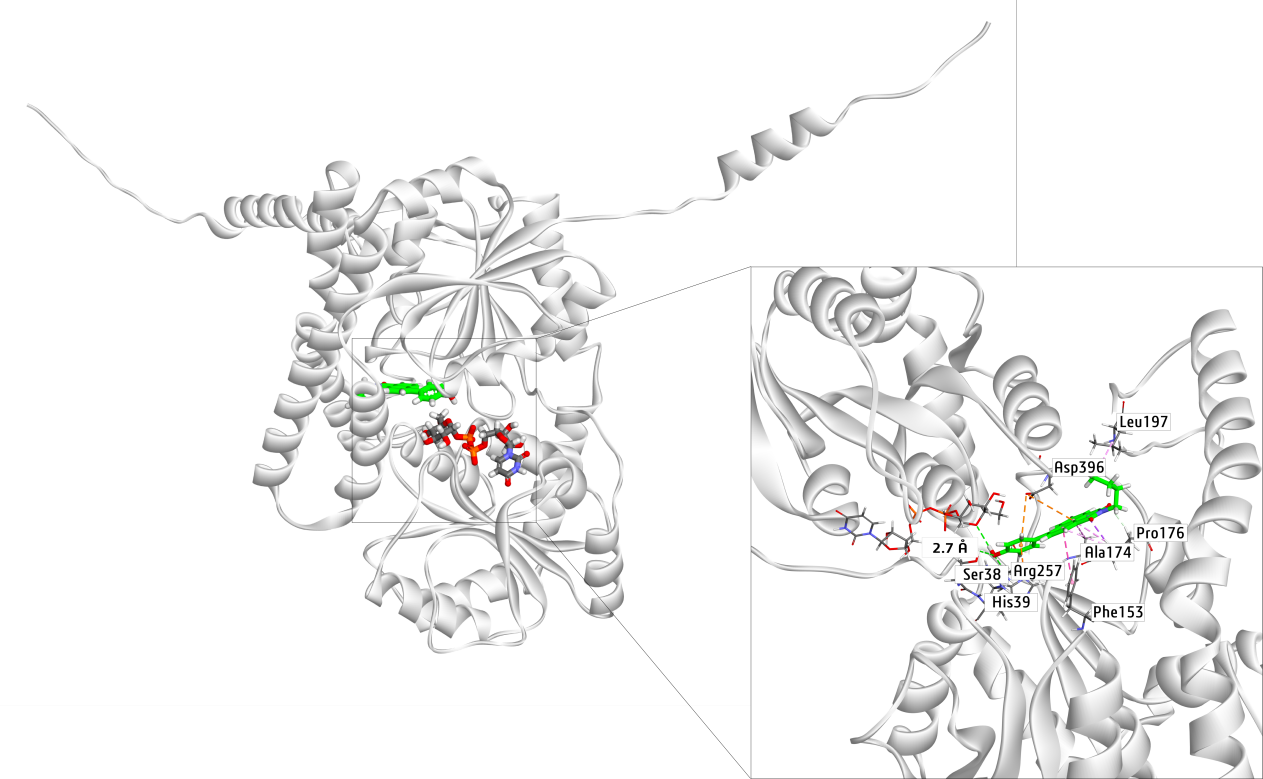


**Fig. S8** The docking simulations of NHPN (light green) in hUGT1A1 complexed with the sugar donor UDPGA (black). The 3D-structure of hUGT1A1 is predicted by the AlphaFold (UniProt code: P22309).


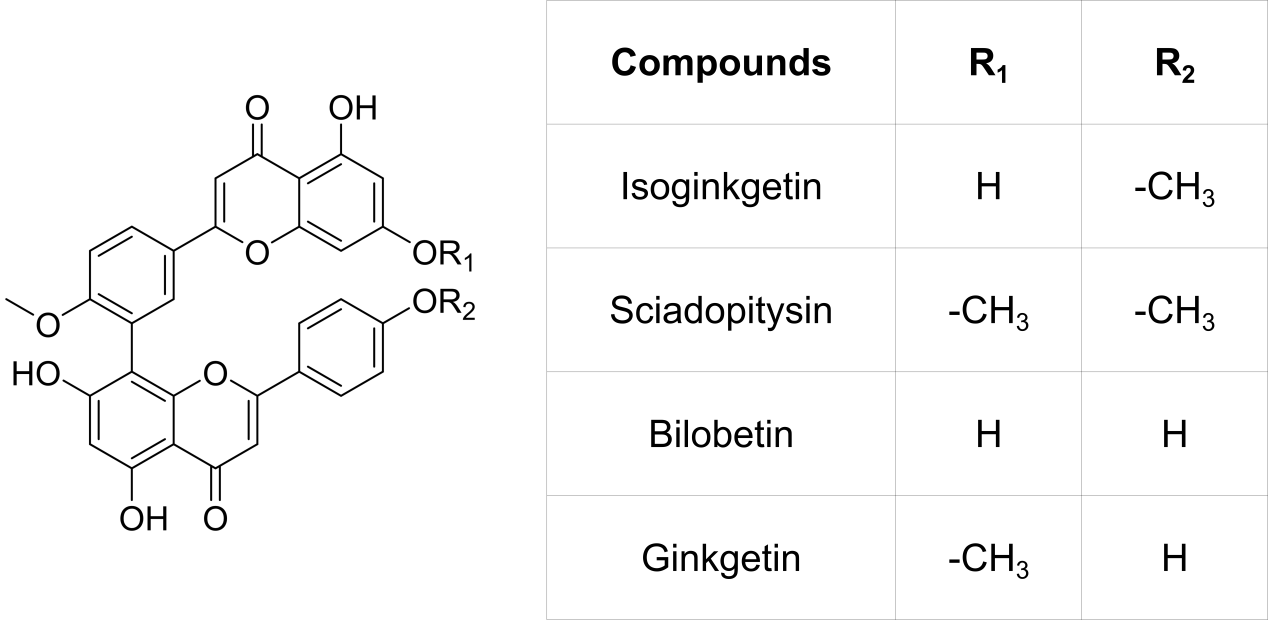


**Fig.S9** Chemical structures of isoginkgetin, sciadopitysin, bilobetin and ginkgetin.


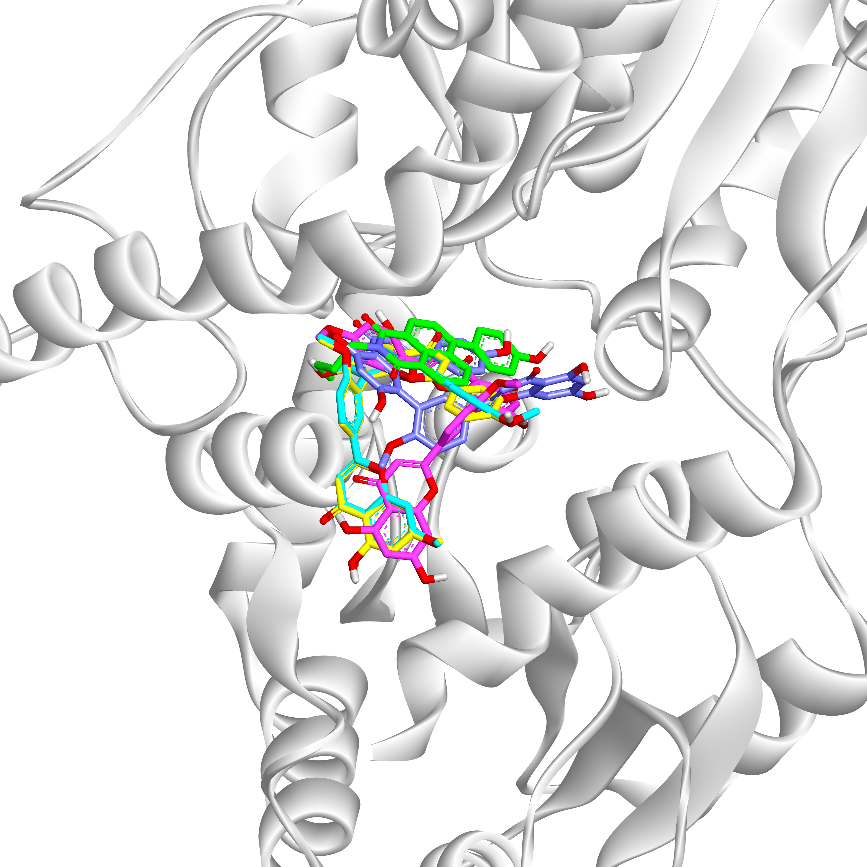


**Fig. S10** Four biflavones (bilobetin in purple, ginkgetin in yellow, isoginkgetin in magenta and sciadopitysin in cyan) highly overlapped NHPN (light green) in the catalytic pocket.


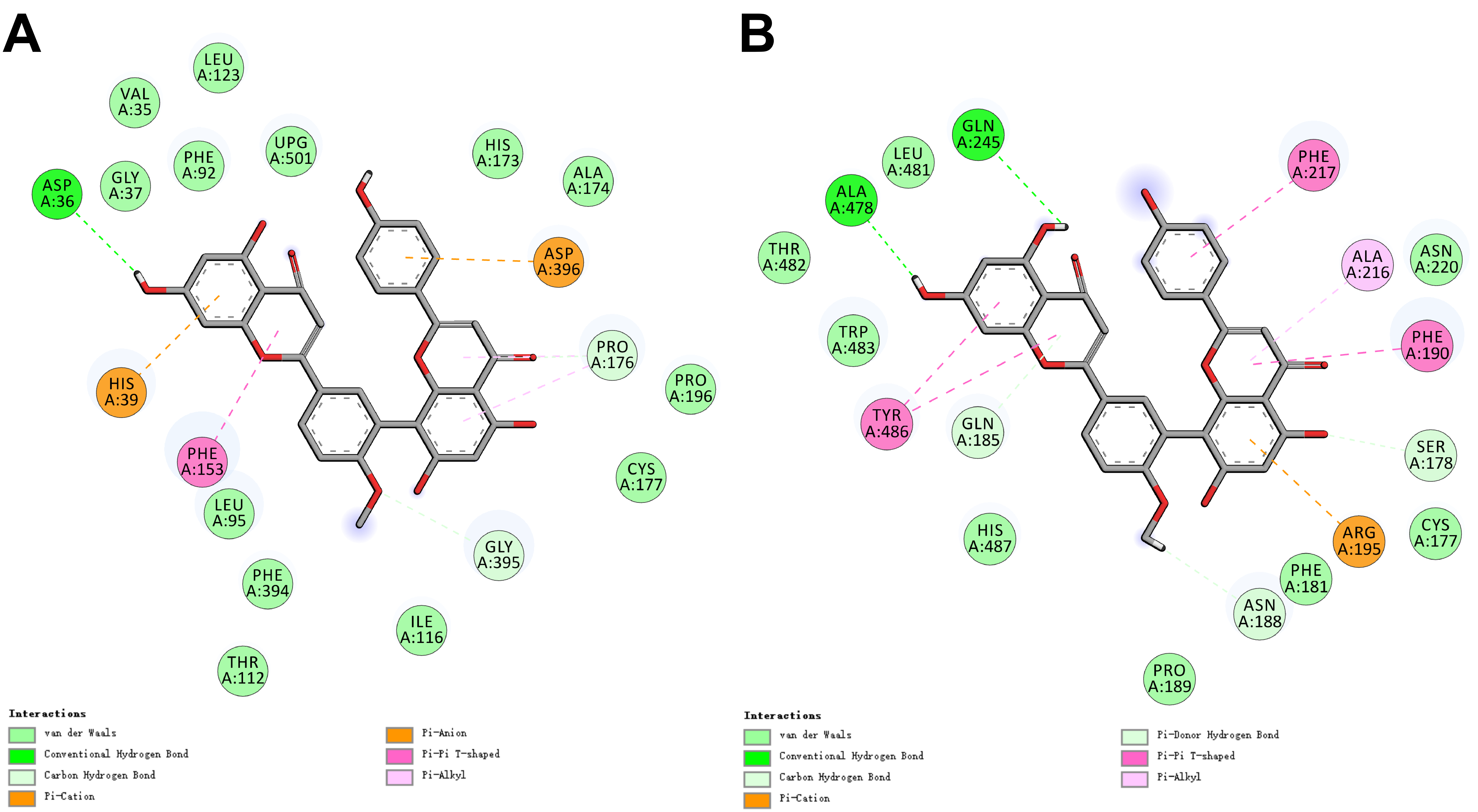


**Fig. S11** The 2D interaction analysis of bilobetin binding on hUGT1A1. A) catalytic site, B) allosteric site.

**
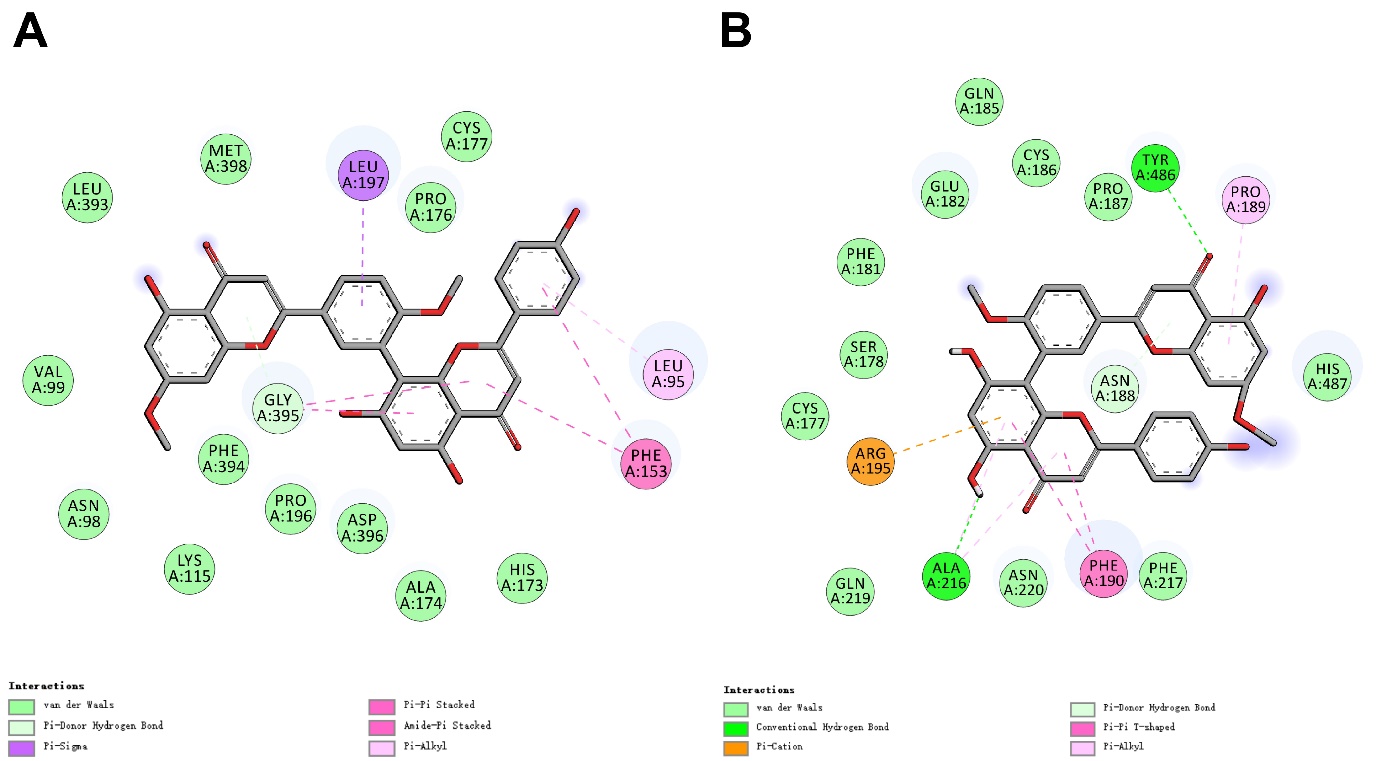
**

**Fig. S12** The 2D interaction analysis of ginkgetin binding on hUGT1A1. A) catalytic site, B) allosteric site.


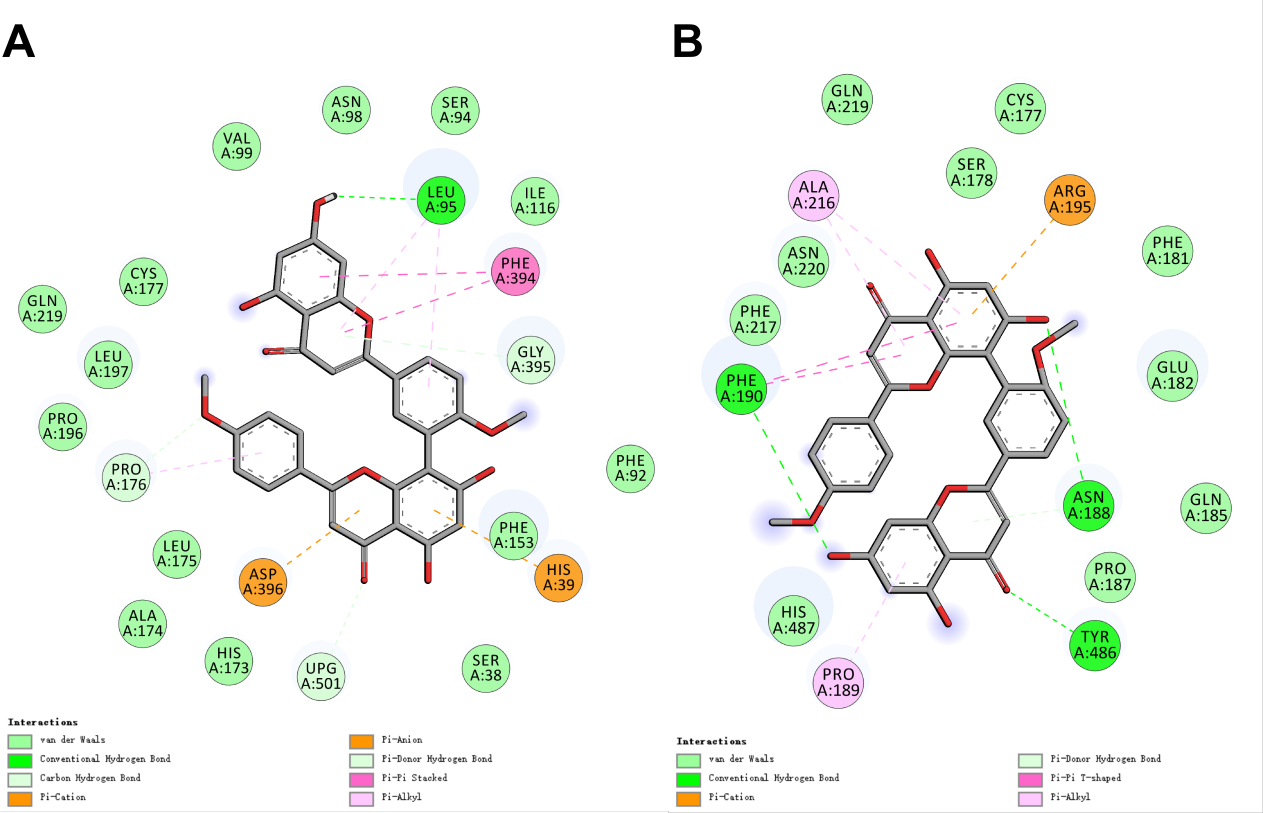


**Fig. S13** The 2D interaction analysis of isoginkgetin binding on hUGT1A1. A) catalytic site, B) allosteric site.


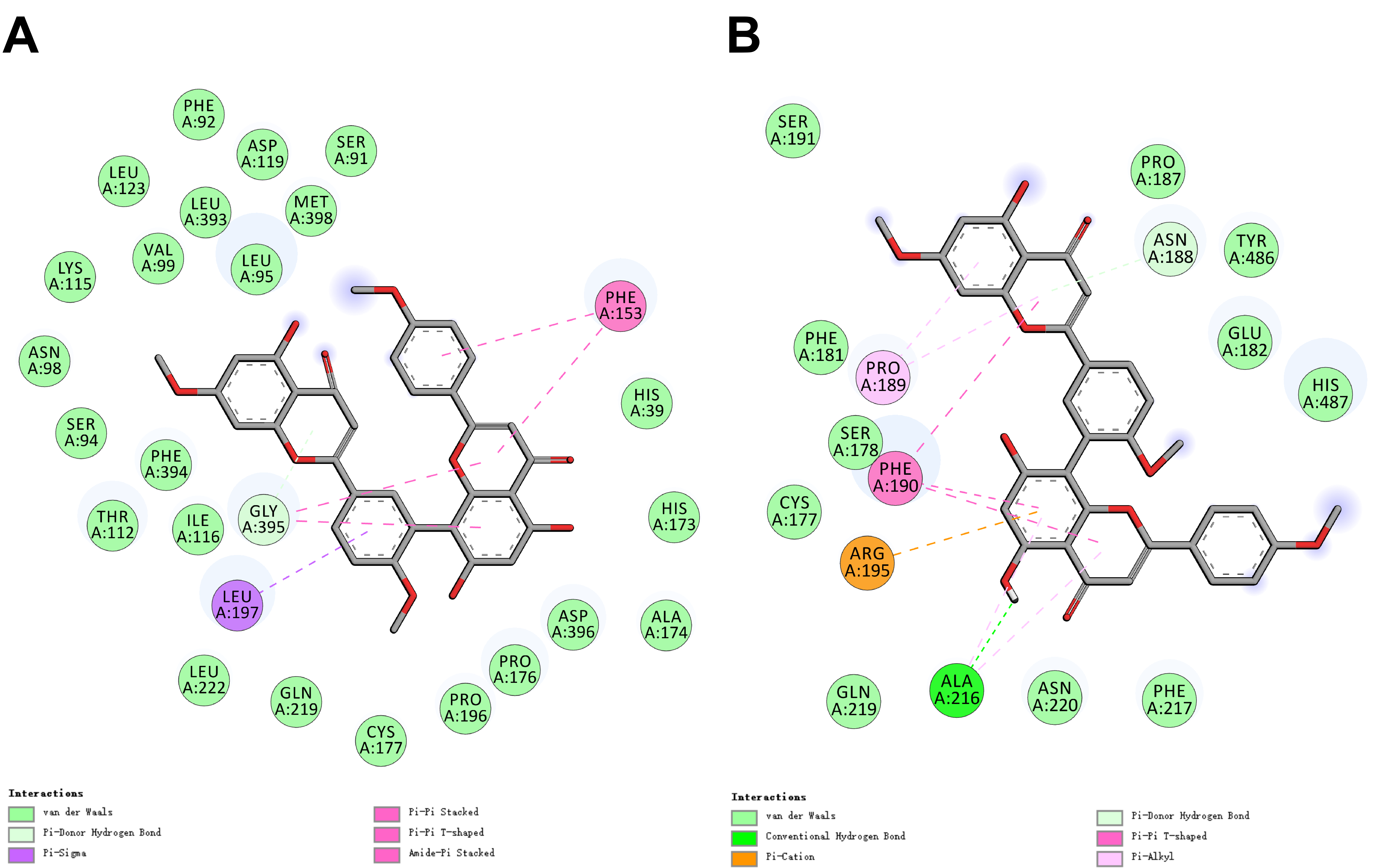


**Fig. S14** The 2D interaction analysis of sciadopitysin binding on hUGT1A1. A) catalytic site, B) allosteric site.


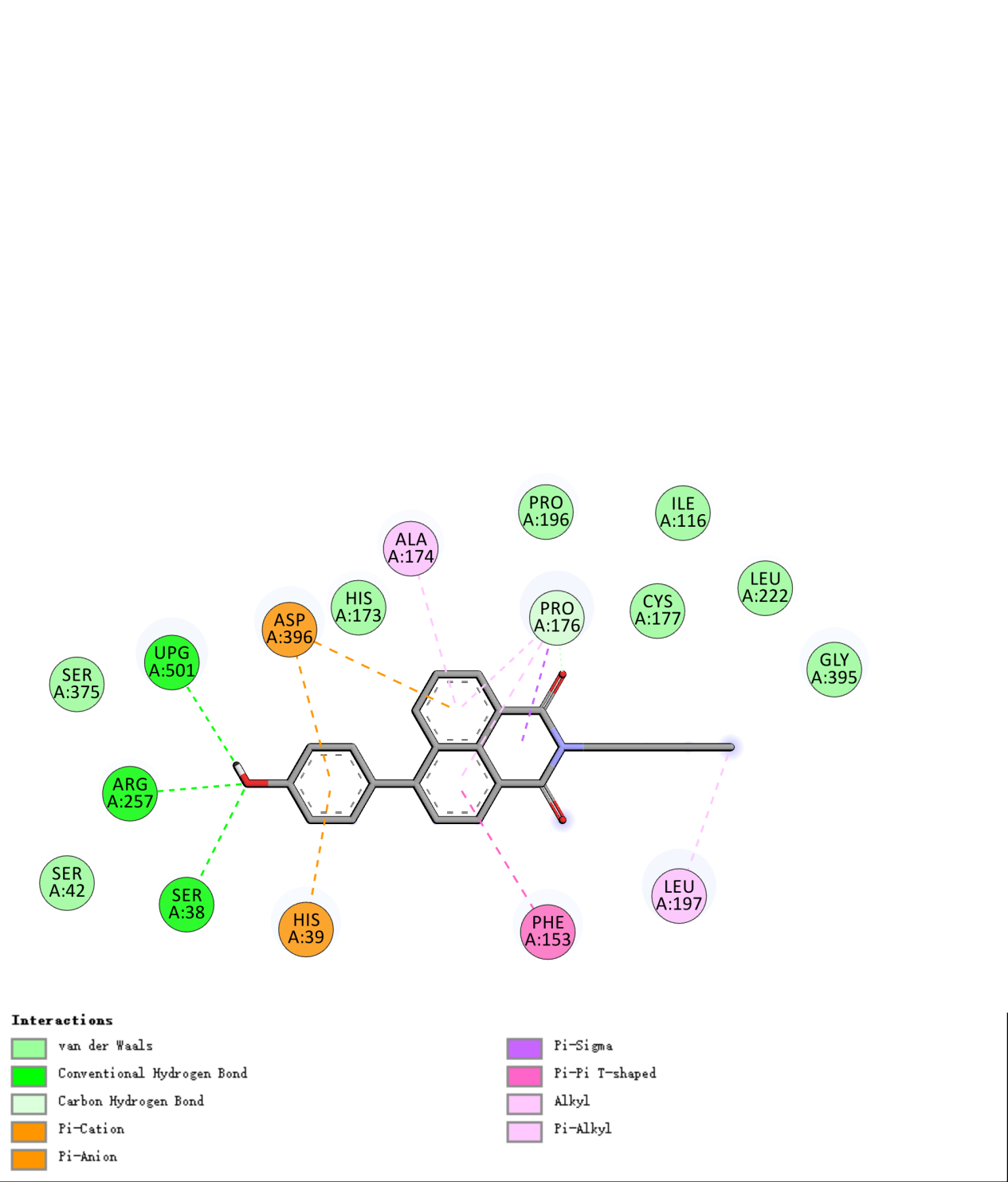


**Fig. S15** The 2D interaction analysis of NHPN binding on hUGT1A1 in the catalytic site.
